# Hybrid Impedimetric Biosensors for Express Protein Markers Detection

**DOI:** 10.3390/mi15020181

**Published:** 2024-01-25

**Authors:** Nikita Sitkov, Andrey Ryabko, Vyacheslav Moshnikov, Andrey Aleshin, Dmitry Kaplun, Tatiana Zimina

**Affiliations:** 1 Department of Micro and Nanoelectronics, Saint Petersburg Electrotechnical University “LETI”, 197022 Saint Petersburg, Russia; vamoshnikov@mail.ru (V.M.); tmzimina@gmail.com (T.Z.); 2 Engineering Centre for Microtechnology and Diagnostics, Saint Petersburg Electrotechnical University “LETI”, 197022 Saint Petersburg, Russia; 3 Laboratory of Nonequilibrium Processes in Semiconductors, Ioffe Institute, 26 Politekhnicheskaya, 194021 Saint Petersburg, Russia; aleshin@transport.ioffe.ru; 4 Artificial Intelligence Research Institute, China University of Mining and Technology, 1 Daxue Road, Xuzhou 221116, China; dikaplun@etu.ru; 5 Department of Automation and Control Processes, Saint Petersburg Electrotechnical University “LETI”, 197022 Saint Petersburg, Russia

**Keywords:** impedimetric biosensors, electrochemical impedance spectroscopy, proteins, nanomaterials, express detection, microfluidics, antibodies, aptamers, peptides, label-free detection

## Abstract

Impedimetric biosensors represent a powerful and promising tool for studying and monitoring biological processes associated with proteins and can contribute to the development of new approaches in the diagnosis and treatment of diseases. The basic principles, analytical methods, and applications of hybrid impedimetric biosensors for express protein detection in biological fluids are described. The advantages of this type of biosensors, such as simplicity and speed of operation, sensitivity and selectivity of analysis, cost-effectiveness, and an ability to be integrated into hybrid microfluidic systems, are demonstrated. Current challenges and development prospects in this area are analyzed. They include (a) the selection of materials for electrodes and formation of nanostructures on their surface; (b) the development of efficient methods for biorecognition elements’ deposition on the electrodes’ surface, providing the specificity and sensitivity of biosensing; (c) the reducing of nonspecific binding and interference, which could affect specificity; (d) adapting biosensors to real samples and conditions of operation; (e) expanding the range of detected proteins; and, finally, (f) the development of biosensor integration into large microanalytical system technologies. This review could be useful for researchers working in the field of impedimetric biosensors for protein detection, as well as for those interested in the application of this type of biosensor in biomedical diagnostics.

## 1. Introduction

Proteins are vitally important biomolecules, present in all living organisms, which form many essential biological compounds such as enzymes, hormones, antibodies, etc., that are involved in various vital processes in the body, such as catalysis, transport, signaling, regulation, defense, structure maintenance, and so on. Proteins also could serve as biological markers of diseases, that is, molecules indicating the presence or absence of normal or pathological conditions in the body. That is why the detection and quantification of proteins in biological fluids is very important for the diagnosis and monitoring of various diseases such as cancer, infections, autoimmune diseases, allergies, etc. [1,2,3,4,5,6,7,8]. It is noted that for accurate and timely identification of the disease, it is necessary to determine a diagnostically important group of protein markers [9,10,11], which allows specialists to choose efficient treatment tactics. There are a number of methods for determining proteins including enzyme-linked immunosorbent assay (ELISA), fluorescence immunoassay (FIA), surface plasmon resonance (SPR), chromatography, spectroscopy, electrophoresis, etc. [12,13]. However, at present, these methods are mostly implemented in the form of stationary laboratory instruments, which have a number of disadvantages, such as relatively high cost, complex and multistage procedures, the need for specialized equipment and reagents for sample preparation, and the use of labels that can affect the properties and activity of proteins. Therefore, there is a need to develop new devices for detecting proteins that would be simpler, faster, more sensitive, and selective. In addition, at present, it is quite difficult to miniaturize these techniques in order to create low-cost portable devices for the rapid diagnosis of diseases at the point of care. These problems can be solved using the biosensing technologies approach.

Biosensors are analytical devices that use specific biochemical reactions to detect chemical compounds, usually using electrical, thermal, or optical detection [14,15,16]. In addition to medical diagnostics, biosensors are also used in ecology, the food industry, and agriculture, as they allow for fast, sensitive, selective, and cheap analysis of biological samples. The accuracy of the information obtained from biosensors depends on the composition of the analyte, the biologically active component, the design of the biosensor, and the physical characteristics of the signal transducer. Biosensors play an important role in biomedical research, as they can provide diagnosis and monitoring of various diseases, as well as study the mechanisms of biological processes at the molecular and cellular levels [17].

In the classification of biosensors according to the principles of signal transduction, electrochemical biosensors occupy a special place, since they have a number of advantages, such as simplicity of design, the possibility of miniaturization and automation, as well as a low level of interference from the environment [18]. They have the potential to provide continuous monitoring and can be implanted into the human body [19]. Electrochemical biosensors can be classified according to the type of electrical parameter being measured into potentiometric, amperometric, and impedimetric [20]. Impedimetric biosensors are based on measuring changes in the electrical impedance of an electrochemical cell upon binding of biomolecules to the surface of electrodes that are modified with specific biorecognition elements. Unlike amperometric and potentiometric biosensors, impedimetric biosensors do not require labels to detect the analyte [21,22], which significantly reduces the cost and simplifies their production.

**Figure 1:**
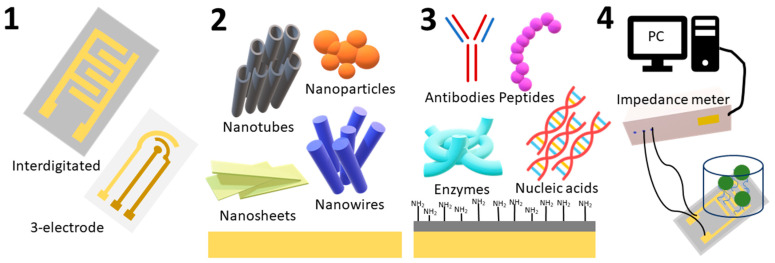
A general scheme for the development of a hybrid impedimetric biosensor for protein detection. 1—selecting the design and material of electrodes; 2—electrode modification and functionalization; 3—selection and immobilization of biorecognition element; 4—implementation of the interface with measurement circuit and sample analysis system.

Impedimetric biosensors have a number of characteristics that make them suitable for protein detection. Firstly, they allow the measurement of frequency dependencies of electrical impedance, which correlates with various parameters of the binding process of proteins and biorecognition elements of the sensor, such as kinetics, thermodynamics, mass transfer, and structural changes to be carried out [23]. Secondly, they allow electrodes to be modified with various nanomaterials and nanostructures, which increase the surface area of the electrodes, improve electron transfer, and enhance the biosensor signal [24]. Thirdly, they allow the use of different types of biorecognition agents, such as antibodies, aptamers, peptides, receptors, or enzymes, which provide specific and stable binding to target proteins [25,26]. Fourthly, they allow the creation of miniature hybrid integrated biosensor matrices coupled with microfluidic systems that reduce sample and reagent volumes, increase the speed and accuracy of analysis, and implement multichannel and parallel measurement [27]. Fifthly, they allow the use of new signal processing, information technology, and artificial intelligence techniques to improve resolution, reduce noise, optimize parameters, classify data, and automate analysis [28]. A general scheme of the hybrid impedimetric biosensor for protein detection development is presented in Figure 1.

At the same time, the development of new impedimetric biosensors for detecting proteins poses a number of challenges for researchers, such as (i) the selection of optimal materials and nanostructures for electrodes, (ii) the development of effective methods for immobilizing biorecognition elements for the formation of stable specific binding groups on the surface of electrodes, (iii) increasing specificity and sensitivity of biosensors, (iv) reducing the influence of interference and nonspecific binding, (v) adapting biosensors to real samples and operating conditions, and (vi) expanding the spectrum of detected proteins. To successfully solve these problems, it is necessary to apply an interdisciplinary approach, combining knowledge and skills in the fields of chemistry, physics, biology, materials science, nanotechnology, electronics, information technology, and medicine. This review will discuss the operation principles of impedimetric sensors, methods of modification and nanomodification of electrode surfaces, types of biorecognition agents and methods of their immobilization, and the integration of impedimetric biosensors into microfluidic systems. We hope that this review will be useful for researchers working in the field of impedimetric biosensors for protein detection, as well as for those interested in the application of such biosensors in biomedical diagnostics.

## 2. Basic Principles of Impedimetric Measurements

Electrochemical impedance spectroscopy (EIS) is a technique that measures the complex impedance, combining the effects of resistance and reactance in a circuit, when an alternating voltage of varying frequency is applied to electrodes. The resistance depends on the physicochemical processes occurring at the electrode–electrolyte interface, such as charge transfer, diffusion, adsorption, desorption, and biological interaction. Impedimetric biosensors belong to label-free biosensors, which are based on a measurement of the impedance value change in the sensor system as a result of the specific binding of the biorecognition element and the target analyte, depending on its concentration. These biosensors are based on two types of EIS: faradaic and non-faradaic. Biosensors for faradaic and non-faradaic EIS have differences not only in the measurement methodology but also in the materials used to modify the electrodes and in design.

**Figure 2:**
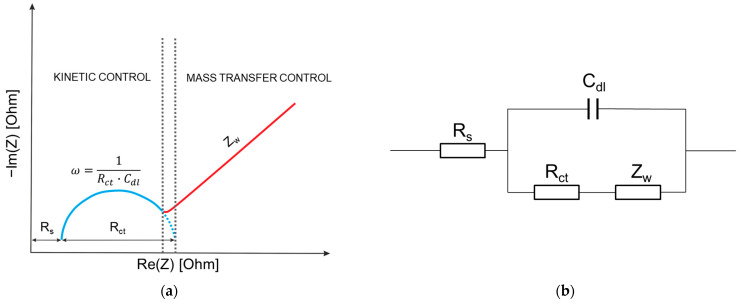
Typical Nyquist diagram (**a**) with corresponding equivalent electrical circuit (**b**).

Faradaic EIS is sensitive to the presence of a redox-active probe in the solution, which participates in an electrochemical reaction on the surface of the electrode. This reaction involves electron transfer between the probe and the electrode, which can be hindered by the binding of the analyte to the bioreceptor. To carry out the faradaic EIS measurements, as a rule, it is necessary to use a redox couple in solution, which is usually a ferro/ferri electrolyte solution (K_3_Fe(CN)_6_ + K_4_Fe(CN)_6_·3H_2_O) [29,30,31]. Before performing EIS, biosensors are usually immersed in a solution (or biological fluid) for immobilization of the target analyte, usually washed, and then immersed in the solution for measurements [32,33,34]. The electrochemical cell, in addition to the biosensor electrode, also contains a counter electrode (CE) and a reference electrode (RE). The biosensor plays the role of a working electrode (WE). A typical faradaic EIS spectrum is presented in Figure 2a as a Nyquist plot. It contains a semicircle at high frequencies due to the charge transfer resistance (R_ct_) associated with the oxidation of the redox pair and the capacitance of the electrical double layer (C_dl_). Also, the Nyquist diagram contains a straight line at low frequencies, which is associated with mass transfer, including diffusion [35].

Thus, the electrochemical system for faradaic EIS is typically represented as a Randle equivalent circuit, which contains the electrolyte resistance R_s_ between the biosensor (WE) and the reference electrode (RE), the electrical double-layer capacitance C_dl_ (or constant phase element (CPE)), charge transfer resistance R_ct_, and the Warburg element (Z_W_) (Figure 2b). Charge transfer resistance (R_ct_) is the most important parameter for faradaic EIS, as it reflects the activation barrier to electron exchange between the redox probe and the electrode. As shown in Figure 2a, the diameter of the semicircle is determined by the value of the charge transfer resistance R_ct_, the change in which is used to construct the concentration dependence of the biosensor signal [36,37,38].

To ensure a good level of electronic conductivity between the working electrode and the ferri–ferro electrolyte, it is useful to provide a sufficiently low charge transfer resistance R_ct_ for the initial electrode [39,40,41]. The binding of biorecognition molecules (for example, anti-CYFRA 21-1) to the nanocomposite leads to an increase in R_ct_. Blocking nonspecific binding sites with bovine serum albumin (BSA) further reduces the electron transfer process between the working electrode and the electrolyte [42]. Finally, specific immobilization of the analyte leads to a significant blocking of charge transfer for the oxidation of the redox pair and, accordingly, an increase in R_ct_. Therefore, the analyte concentration significantly affects the diameter of the semicircle on the Nyquist diagram. Thus, it is possible to set up a calibration concentration dependence on the change in R_ct_. It can be linearly approximated, which is quite convenient for automating the determination of biomarker concentration. However, to determine R_ct_, preliminary mathematical processing of Nyquist diagrams and spectroscopy in a wide frequency range of alternating voltage is necessary.

It should also be noted that the electrochemical system for faradaic EIS allows a cyclic voltammetry (CV) measurement to be carried out. During this process, when the analyte concentration increases, the peak current values decrease. However, concentration dependences are usually determined relative to ΔR_ct_. The initial modified working electrode of the biosensor must have sufficiently good electrical conductivity to ensure the process of charge transfer between the electrode and the electrolyte; therefore, conductive materials are used in the formation of the electrode or electrode-modifying material [43,44,45].

Unlike faradaic EIS, non-faradaic EIS does not use redox reactions, that is, measurements are carried out in a buffer solution or biological fluid without a specific electrolyte and without additional reference (RE) and counter (CE) electrodes. The processes of analyte immobilization and spectroscopy are often carried out in the same solution [46,47,48,49]. In non-faradaic EIS, there is no electrochemical reaction on the electrode surface, but only the formation of an electrical double layer, which can be modeled as a capacitor. The double-layer capacitance (C_dl_) depends on the dielectric properties of the medium, the electrode area, and the distance between the capacitor plates. When the analyte binds the biorecognition element on the electrode surface, these parameters change, which leads to a change in the capacitance of the double layer. Thus, non-faradaic EIS measures the change in capacitance between the double layer and the electrode interface, which reflects the change in the dielectric behavior of the sensor surface. The biosensor itself, instead of one electrode, must contain two electrodes (for example, interdigitated once, increasing the area between the electrodes) between which there is a material to which immobilization is ensured. Dielectric and high-resistivity semiconductor materials are used as such materials.

For non-faradaic EIS, it is necessary to use an insulating layer on the electrode to prevent electrochemical reactions and make the interface capacitance the dominant parameter. For an ideal insulator or in the absence of redox compounds, the leakage resistance is theoretically infinite [50]. In order for this resistance to tend to infinity in practice, the electrodes can be electrically isolated from the immobilization material. Thus, in [51], a ZnO/CuO nanofilm is separated from the electrodes by borosilicate glass.

**Figure 3:**
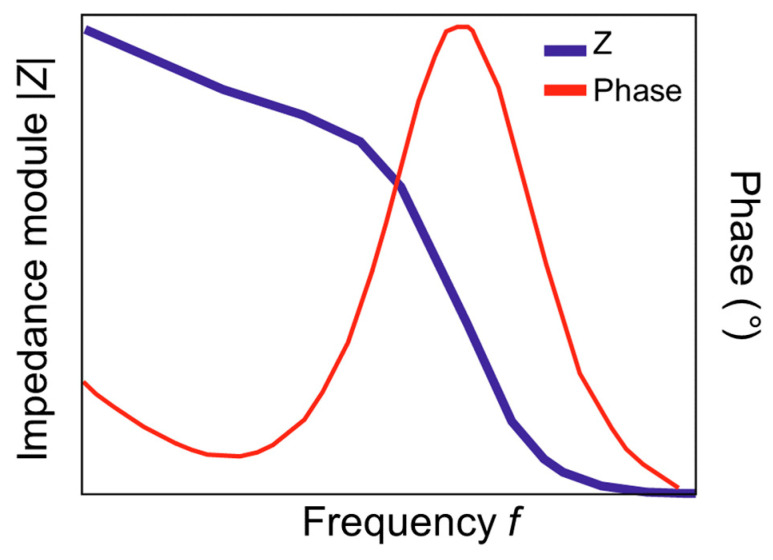
Schematic example of a Bode plot for impedance module, |Z|, and phase shift versus frequency, *f*.

Non-faradaic EIS spectra are usually presented as Bode diagrams, which show the dependence of the impedance modulus (|Z|) and phase shift (φ) on the logarithm of frequency (Figure 3). For non-faradaic EIS, when there is no redox-active probe in the solution, the Bode diagram is preferable, since it allows one to study the frequency dependence of the impedance. In addition, other parameters such as capacitance (C) and conductance (Y) can be used to represent non-faradaic EIS results. In general, the Bode diagram also makes it possible to identify the frequency at which it is preferable to determine the biosensor response. When choosing such a frequency, you can proceed, for example, from the maximum signal-to-noise ratio [52,53]. The ability to use only one frequency in non-faradaic impedimetric biosensors is very important for their practical application since this significantly simplifies the required equipment when used in the analysis [54].

In general, it should be noted that non-faradaic impedimetric biosensors can be much simpler for practical use, since they do not require additional electrodes or the addition of a redox pair and can operate at a single frequency, while faradaic biosensors, as a rule, analyze over a fairly large frequency range and require a slightly more complex preparation (electrochemical cell). However, faradaic impedimetric biosensors can generally demonstrate significantly greater analytical sensitivity. Also, for a general overview of faradaic and non-faradaic label-free impedance biosensors, one can refer to the review articles [50,55,56].

## 3. Materials for Electrodes and Their Modifications

Impedimetric biosensors can be made from various materials, both to form the electrodes and to modify them. In faradaic EIS biosensors, in addition to the working electrode, a counter electrode and a reference electrode are also used. To create a working electrode, which, in fact, is the sensor itself, various materials are used, both for the conductive electrode and its modification for subsequent functionalization. In non-faradic EIS biosensors, two electrodes are used for measurements, between which another material is located, serving for subsequent functionalization. In this section, we will highlight the main groups of materials for the formation of electrodes, which are directly functionalized to create an impedimetric biosensor. We will also review some examples of impedimetric biosensors from the main groups of electrode materials.

### 3.1. Metals

Impedimetric biosensors with a metal working electrode typically contain gold electrodes that are functionalized with molecules to form self-assembled monolayers (SAMs) for subsequent binding [57,58,59,60,61,62,63,64,65,66,67,68].

**Figure 4:**
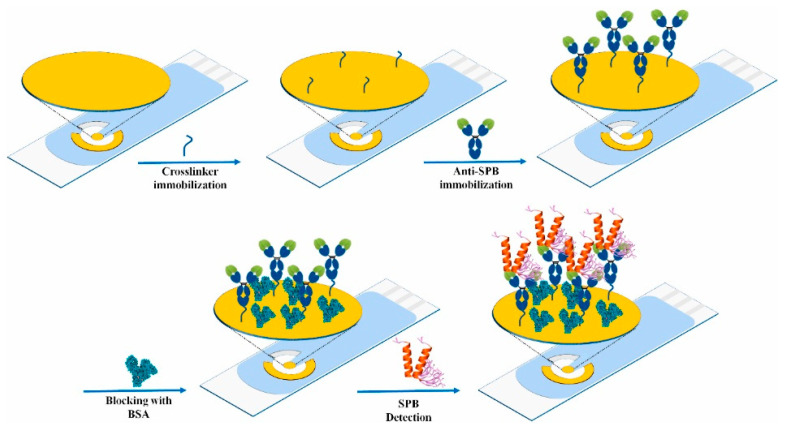
Schematic presentation of immunosensor preparation process for SPB detection. Reprinted from [68] with permission of Elsevier provided by Copyright Clearance Center.

In [68], an impedimetric immunosensor was developed based on gold screen-printed electrodes (SPE, DropSens, Oviedo-Asturias, Spain) for the detection of surfactant protein B (SPB) in human amniotic fluid (AF) samples. A gold electrode was used as CE, and a pseudo-reference electrode made of silver was used as RE. The biofunctionalization of the working electrode (biosensor electrode) is presented in Figure 4. 

The surface of the gold electrode was precleaned in isopropanol and deionized water and dried under N_2_ flow. The gold surface was incubated in Sulfo-LC-SPDP (sulfosuccinimidyl 6-(3′-(2pyridyldithio)propionamido)hexanoate) to form a SAM. Anti-SPB antibodies were immobilized for 2 h at room temperature. Finally, the surface was incubated in BSA solution (1%) for 20 min to block the exposed groups to prevent nonspecific binding. SPB solutions of varying concentrations were applied to the immobilized electrode for 90 min at room temperature to capture the antigen. After thorough washing, they were placed in redox probe solution (5 mM [Fe(CN)_6_]^3−/4−^ in PBS). The authors demonstrated that with increasing antigen concentration, R_ct_ increases, i.e., the process of charge transfer is reduced. The immunosensor showed a linear dynamic range from 2 ng/mL to 2000 ng/mL and a limit of detection (LOD) of 0.1 ng/mL.

It should be noted that the mass production of such miniature measuring electrochemical platforms makes possible the widespread use of impedimetric biosensors and also simplifies and speeds up their development. For example, a similar sensor platform with gold SPE (Metrohm DropSens, Oviedo-Asturias, Spain) was used in [65] to develop an impedimetric biosensor for Alpha-1-B glycoprotein (A1BG), a marker of endometriosis. To form SAMs on a gold electrode, 3-Mercaptopropionic acid (3-MPA) was used in this work. The SAM was then stirred into the mixture of 1-Ethyl-3-(3-dimethylaminopropyl)carbodiimide (EDC) and *N*-Hydroxysuccinimide (NHS) to allow conversion of the terminal carboxyl group (-COOH) of the SAM to the NHS transition ester.

In [57], interdigitated microelectrode (IDWμE) arrays were used for functionalization with a self-assembled monolayer of thioctic acid (TA) to immobilize the antigen. The developed immunosensor was aimed at detecting rheumatoid factor–immunoglobulin M (IgM-RF). For surface modification, the IDWμEs were immersed in a solution of TA in ethanol, where a SAM-TA was formed by adsorption on the electrodes. After modification of the TA-SAM electrodes, they were washed to remove unbound thiol moieties. The covalent immobilization of human IgG-Fc fragments was carried out to bind the target analyte. The sensor showed an LOD of 0.6 IU/mL in the presence of a redox probe and 0.22 IU/mL in human serum.

Gold, as a material for immobilization, is also used in the form of nanoparticles, which can provide an additional increase in the specific surface area of the electrode [69,70,71,72]. Basic examples of impedimetric biosensors using metal electrodes are given in Table 1.

### 3.2. Conductive Oxides

It is obvious that, in addition to metals, a wide class of conductive materials can be used to make electrodes, including transparent conductive films based on metal oxides [73,74,75,76,77,78,79]. Transparent conductive oxides (TCO) are widely used for solar panels, touch screens, and displays. Among the TCOs, the most widely used are indium tin oxide (ITO) and second-doped tin oxide (FTO). Less commonly used are zinc oxides doped with aluminum or gallium (AZO, GZO). Transparent conductive oxides are used in the production of impedimetric biosensors using the same method as metal ones, that is, SAMs are formed on the surface from molecules containing functional groups. However, their binding to TCO is carried out via hydroxyl groups on the surface.

**Figure 5:**
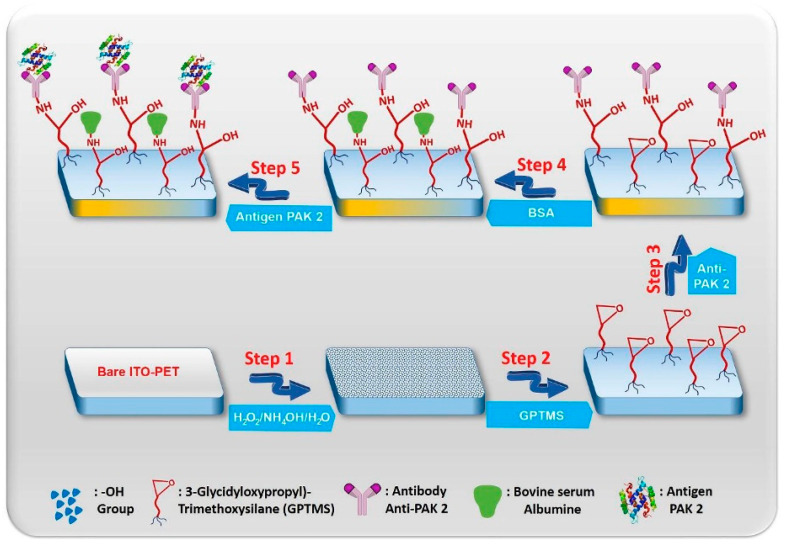
Scheme of an immunosensor for PAK 2 based on an ITO electrode. Reprinted from [76] with permission of Elsevier provided by Copyright Clearance Center.

A typical example of the functionalization and immobilization of a TCO-based electrode, which was developed for the detection of p21-activated kinase 2 (PAK 2), is presented in Figure 5 [76]. The first step is to treat the ITO electrode to form hydroxyl groups on the surface. Then, the surface was silanized in a solution of 3-glycidoxypropyltrimethoxysilane (GPTMS), forming a monolayer connected to the electrode through Si-O-Si bonds. The next steps include immobilization of the recognition layer using anti-PAK 2 and blocking unbound groups with BSA. The final step of the process presented in Figure 5 is the binding of the analyte to the immunosensor. The immunosensor had a linear calibration curve of response to PAK 2 capture in the range of 0.005–0.075 pg/mL with an LOD of 1.5 fg/mL. The immunosensor demonstrated high stability, good reproducibility, and stability.

The binding of SAM to OH groups is also carried out via the formation of P-O bonds. Thus, using an ITO electrode, an impedimetric immunosensor was developed to detect the cancer marker interleukin-8 (IL-8) in human serum and saliva samples [73]. Prewashed ITO-coated substrates were immersed in a solution of NH_4_OH:H_2_O_2_:H_2_O (1:1:5) for 90 min to form hydroxyl groups on the ITO surface. The hydroxylated ITO electrode was then functionalized with a carboxyl-terminated phosphonic acid (PHA) SAM. At this stage, the end groups of SAM-PHA were covalently bonded to the hydroxyl groups, and P–O bonds were formed. The biorecognition layer was formed using immobilization of biorecognition elements, anti-IL8 antibodies, via the carboxyl groups of SAM-PHA, which were chemically activated using EDC/NHS. EDC and NHS acted as a coupling agent and an activator, respectively. After immobilization with anti-IL8, BSA was used to block the carboxyl groups of PHA and other proteins. The impedimetric biosensor showed a wide detection range of IL8 from 0.02 pg/mL to 3 pg/mL and an LOD of 6 fg/mL.

Similarly, a biosensor for the detection of interleukin 1α (IL-1α) was developed in [74], where the surface of FTO was modified with carboxyalkylphosphonic acid (PHP) and immobilized with anti-IL-1α antibodies. IL-1α antigen concentration was determined over a linear detection range of 0.02 to 2 pg/mL. The LOD value of the proposed immunosensor was 6 fg/mL. The FTO electrode surface was also hydroxylated with NH_4_OH/H_2_O_2_/H_2_O solution. The FTO electrode was then immersed in PHP solution to form a layer, and after washing, the carboxyl groups in the NHS/EDC solution were activated. Immobilization was also carried out by immersing the electrodes in a phosphate-buffered solution containing antibodies to IL-1α. Anti-IL-1α binding is mediated by the amino groups of antibodies and the carboxyl groups of PHP.

In [79], a coating was applied to vertically oriented silicon nanowires to increase the specific area of an impedimetric biosensor and to expand the concentration range of ITO. This gave the linear detection range of human cardiac troponin I (cTnI) in the range from 1.760 to 1760 ng/mL.

Basic examples of impedimetric biosensors using conductive oxides are given in Table 2.

### 3.3. Carbon Materials

Various carbon materials have good electrical conductivity and are widely used as materials to produce electrodes like TCO electrodes, Au electrodes, and other metal electrodes. Electrodes based on carbon materials are also applicable for chemical modification of the surface to provide selective coupling of analytes. And since carbon-based nanomaterials not only have good electrical conductivity but also provide a high specific surface area and the possibility of modification, they are also often used to form nanocomposites. This section will review the applications of various carbon materials with chemically modified surfaces for analyte biorecognition.

Carbon electrodes, as in the case of Au electrodes, are applied using the screen-printing method [80,81,82]. Moreover, to increase the specific area, screen-printed carbon electrodes (SPCE) based on graphite can be modified with carbon nanotubes [81,82,83,84]. It should be noted that other types of electrodes could also be modified with carbon nanotubes, such as, for example, the gold electrodes described in [85] or the ITO electrodes described in [86].

Senturk et al. [81] presented an impedimetric aptasensor for the detection of lysozyme (LYZ), in which the peptide aptamer was immobilized on the surface of screen-printed electrodes made of carbon nanofibers (DropSens, Oviedo-Asturias, Spain). During the passive adsorption of reagents, the formation of peptide bonds between the carboxyl groups of CNF and the NH_2_ groups of the aptamer occurred. When the aptamer complexed with LYZ, the reaction blocks electron transfer at the interface. Thus, an increase in concentration led to an increase in R_ct_, from which the quantitative response of the biosensor was determined. Under optimal conditions, the LOD of the aptasensor is 0.36 μg/mL. The aptasensor was also used to determine LYS in fetal serum samples of cattle with an LOD value of 1.89 μg/mL. 

In [87], a working electrode made of multi-walled carbon nanotubes was modified with graphene quantum dots and gold nanoparticles (AuNPs@MWCNTs-GQDs). This working electrode was used to create an impedimetric immunosensor for the detection of a prostate cancer biomarker in human serum—a prostate-specific antigen (PSA). Activation of the carboxylate group of the nanocomposite was carried out in a solution of EDC and NHS. The AuNPs@MWCNTs-GQD electrode was incubated for antibody binding for 45 min. Blocking of nonspecific binding sites was performed using BSA. Impedance gain was linearly related to PSA concentration over a wide range from 1 to 10,000 pg/mL with an LOD of 0.48 pg/mL. The authors note that modification of the electrodes not only leads to an increase in the measured signal level due to an increase in the rate of electron transfer but also increases the ability of the electrode surface to adsorb bioactive substances. Carbon nanotube MWCNTs were also modified with AuNPs in [86].

Glassy carbon [38,48] and graphite paper [88,89,90] are also used as electrodes based on carbon materials. And in [90], graphite paper was modified with AuNPs. In [59], in addition to glassy carbon, boron-doped diamond was also used as an electrode, as well as in [91] for the detection of SARS-CoV-2 S1. 

**Figure 6:**
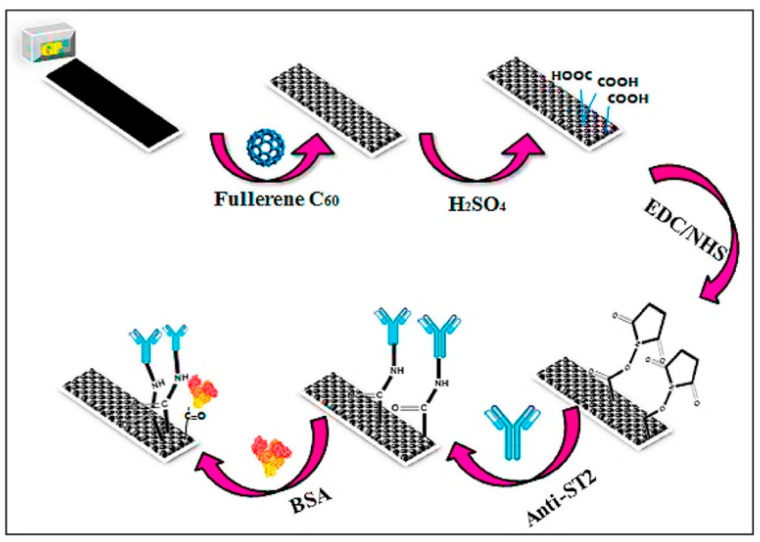
ST2 immunosensor immobilization scheme. Reprinted from [88] with permission of Elsevier provided by Copyright Clearance Center.

In [88], a highly sensitive immunosensor based on a disposable graphite paper (GP) electrode modified with C60 fullerene was developed to determine the suppression of tumorigenicity 2 (ST2) in human serum. GP electrodes were coated with a solution of C60 fullerene (in toluene) and thoroughly dried with pure argon gas. To break the carboxyl groups on the electrode surface, it was incubated with an H_2_SO_4_ solution overnight in the dark. The electrodes were then incubated in EDC/NHS in phosphate buffer for 1 h. To immobilize the electrodes, they were immersed in anti-ST2 solutions at room temperature for 60 min. BSA was used as a blocking agent. The overall design of the ST2 immunosensor is presented in Figure 6. The ST2 impedimetric immunosensor demonstrated excellent repeatability, reproducibility, and a wide detection range (0.1 fg/mL to 100 fg/mL). The LOD and limit of quantitation (LOQ) values were 0.124 fg/mL and 0.414 fg/mL, respectively.

In [92], graphene foam was used as a flexible electrode in a microfluidic laboratory-on-a-chip to detect interleukin 10 (IL-10). In this work, the conductivity of graphene was maintained by noncovalent π–π functionalization with pyrene carboxylic acid (PCA). Graphene foam Gii-sense^®^ (Integrated Graphene Ltd., Eurohouse, UK) electrode working areas were directly grown on polyimide substrates without the use of a catalyst and a transfer process. A solution of PCA in dimethylformamide (DMF) was used to functionalize the working electrode. The electrode was immobilized with a polyclonal anti-IL-10 antibody. Nonspecific sites were blocked with ethanolamine. The PCA antibody modification showed that the graphene foam withstands the incubation, measurement, and washing performed in a microfluidic device. Also, when studying the working electrode using the contact angle method, it was found that the wettability of the graphene surface gradually increased after each modification step. The Gii-sense^®^/PCA/anti-IL-10 biosensor was tested with artificial saliva samples containing various concentrations (from 10 to 100 fg/mL) of IL-10. Automated detection measurements performed in a 3D-printed microfluidic device showed a linear relationship between 10 fg/mL and 100 fg/mL with an LOD value of 7.89 fg/mL in artificial saliva. Specificity was assessed against interleukin 6, TNF-α, and BSA.

Graphene oxide is widely used to modify the electrodes of impedimetric biosensors [93,94,95,96,97,98]. Filip et al. [93] studied the anchoring of the lectin concanavalin A (ConA) on the surface of electrochemically reduced graphene oxide (ErGO) with thionine (Thi) using glutaraldehyde (GA) as a crosslinker and the use of this method for impedance detection of invertase glycoprotein (INV), which has an affinity to ConA. It has been established that the application of ConA/GA complexes to the ErGO/Thi surface allows the biosensor to achieve linear characteristics in the INV concentration range from 10^−14^ to 10^−8^ mol. These results indicated the possibility of attaching biorecognition molecules to weakly reduced or unreduced graphene oxide. In [94], a nanocomposite was prepared from reduced graphene oxide (rGO) and gold nanoparticles (AuNP) to create a disposable neurobiosensory probe for the determination of the Tau-441 protein, which is a marker of Alzheimer’s disease. The surface of the nanocomposite (rGO-AuNP) was modified with 11-mercaptoundecanoic acid (11-MUA), acting as a covalent anchor to improve the sensitivity of the assay. Activation of the end groups was carried out in an EDC/NHS solution. The electrodes were incubated in an anti-Tau solution for 60 min at room temperature. To block nonreactive ends, electrodes were incubated in a BSA solution. The immunoreaction of Tau-441 with anti-Tau was monitored in real time using single-frequency impedance (SFI). The developed immunosensor demonstrated a linear response in the concentration range of 1–500 pg/mL and an LOD value of 0.091 pg/mL.

**Figure 7:**
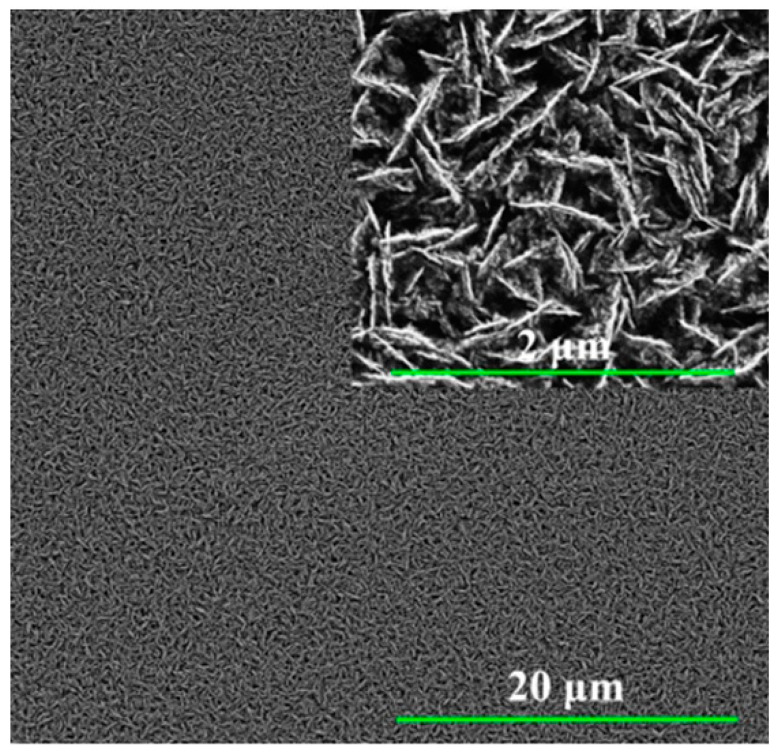
SEM micro-images of the bare B:CNW surface. Reprinted from [99] with permission of Elsevier provided by Copyright Clearance Center.

In addition to the use of boron-doped diamond, a nonstandard carbon nanomaterial was used to create a biosensor in [99]. It reports a new impedimetric immunosensor for the detection of protein *D* in purified and bacterial (*Haemophilus influenzae*, Hi) samples. Recognition was based on the immobilization of anti-protein *D* antibodies (apD) onto maze-like boron-doped carbon nanowall electrodes (B:CNW). The SEM image of the sensor is shown in Figure 7. The B:CNW electrodes were prepared by CVD using diborane (B_2_H_6_) as a precursor for acceptor doping. Other precursors were H_2_ and CH_4_ gases. The resulting B:CNW electrodes were modified in an electrochemical cell in a diazonium salt solution to produce B:CNW/C_6_H_5_COOH. Electrografting on B:CNW was carried out with polarization from 0 V to 1 V 11 times. To immobilize ligands on the B:CNW/C_6_H_5_COOH electrodes, an apD solution together with a carboxyl group activator (EDC/NHS) was applied to the electrode surface. The immunosensor demonstrated a linear antigen detection range. The LOD of protein *D* detection was 2.39 × 10^2^ fg/mL with a linear range from 3.37 × 10^−11^ to 3.37 × 10^−3^ μg/mL (in the purified sample).

**Figure 8:**
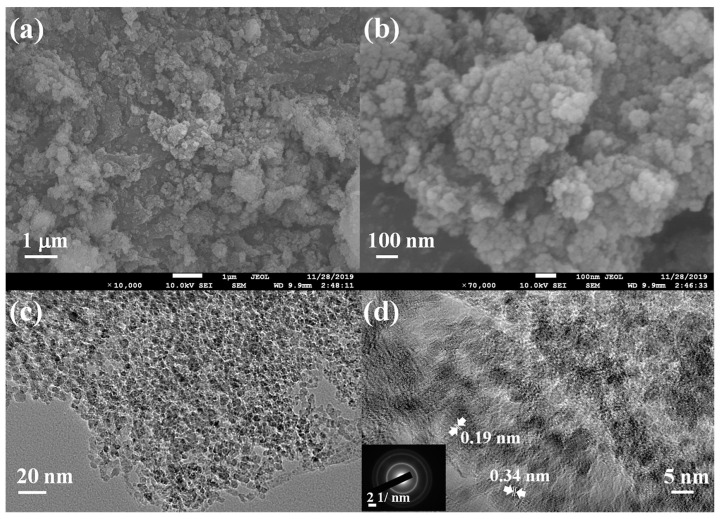
SEM image with low and high magnification (**a**,**b**), TEM (**c**), and high-resolution TEM (**d**) images of HsGDY@NDs. Reprinted from [100] with permission of Elsevier provided by Copyright Clearance Center.

An interesting heteronanostructure (HsGDY@NDs) was obtained by mixing nanodiamonds (ND) and hydrogen-substituted graphdiyne (HsGDY) to create an impedimetric aptasensor for the detection of biomarkers: myoglobin (Myo) and cardiac troponin I (cTnI) [100]. HsGDY@ND (Figure 8) consists of nanospheres of 200–500 nm size, in which NDs are embedded in the HsGDY network. The HsGDY@NDs nanostructure combines the good chemical stability and 3D porous network morphology of HsGDY. The resulting sensor provided an LOD of 6.29 fg/mL and 9.04 fg/mL for cTnI and Myo, respectively, high selectivity, stability, and reproducibility.

Examples of impedimetric biosensors using carbon materials are given in Table 3.

### 3.4. Polymers

Functionalization of conductive electrodes can also be carried out by modifying them with polymers [101,102,103,104,105,106,107,108,109,110]. This approach is often used to modify conductive electrodes directly with macromolecules containing functional groups to form SAMs. In this case, thin polymer layers deposited on the electrodes must have a low enough resistance to allow the charge transfer process to occur for the faradaic EIS mode.

The work [109] describes an impedimetric immunosensor for the determination of CCR4, a biomarker for prostate cancer. The sensor was formed by deposition of acid-substituted poly(pyrrole) P(Pyr-Pac) polymer on an ITO electrode with subsequent immobilization of anti-CC chemokine receptor 4 (CCR4) antibodies. P(Pyr-Pac) contained many carboxyl groups for antibody attachment. To create the ITO sensor, the electrodes were hydroxylated (in a H_2_O:NH_4_OH:H_2_O_2_ solution). Then, the electrode was immersed in a solution of the P(Pyr-Pac) polymer to form self-assembled polymer monolayers with the formation of an ester bond. Immobilization of anti-CCR4 antibodies was achieved using a binding reagent (NHS) and a crosslinking reagent (EDC). The electrode was modified with BSA molecules to block the carboxyl end groups. The developed immunosensor demonstrated a linear EIS response range to CCR4 concentration ranging from 0.02 pg/mL to 8 pg/mL and an LOD of 6.4 fg/mL. A slightly different approach is described in [106], where a polypyrrole polymer containing epoxy active side groups (PPCE) was conjugated to an ITO electrode to detect interleukin 6 (IL-6). For ITO coating, the synthesized pyrrole monomer containing an epoxy active side group (PCE) was subjected to electropolymerization, after which the PPCE-modified electrode was immersed in IL-6 receptor solution and then into BSA solution to prevent nonspecific binding. The biosensor demonstrated a linear range of IL-6 of 0.02–16 pg/mL, an LOD of 6.0 fg/mL, and good selectivity for other interference biomarkers.

Carboxylated polypyrrole was also used to create biosensors in [107,108]. Also described is the use of polypyrrole with epoxy groups in the form of a polymer composite with acetylene black [111]. In [112], overoxidized polypyrrole modified with gold nanoparticles was used. In [113], polypyrrole in the form of nanotubes was used to create a sensor. In addition to polypyrrole, a number of conducting polymers, such as polythiophene [110,114], polyaniline [115], PEDOT [116], and PEDOT:PSS [117], are used to create impedimetric biosensors. At the same time, the small thickness of the polymer layers used to create a biosensor allows the less conductive polymers to be used for modification as well.

In [103], gold electrodes were coated with polytyramine. Polymerization of 25 mM polytyramine in 0.3 M NaOH was carried out on the working electrode using cyclic voltammetry. The potential was varied twice from 0 to 1.6 V and then back to 0 V at a scan rate of 200 mV/s. The resulting polytyramine-modified gold electrodes were used to create an impedimetric biosensor for fibroblast growth factor receptor 3 (FGFR3). To create the biosensor, affimer reagents were isolated, and affimer proteins were synthesized that exhibit strong binding to FGFR. Biotinylated non-affimers were attached to the electrode surface via neutravidin–biotin. The developed impedimetric biosensors showed the ability to detect sub-pM to nM concentrations of recombinant FGFR3 protein in phosphate-buffered solution, as well as in synthetic urine.

A similar architecture of an impedimetric biosensor is presented in [104]. The authors proposed an impedimetric biosensor for the specific detection of Alzheimer’s beta-amyloid oligomers (AβO). The biorecognition element was a fragment of the cellular prion protein (PrPC). The coating of the electrodes in this work was carried out using electropolymerization. To deposit a copolymer of polytyramine/3-(4-hydroxyphenyl) propionic acid (POPA), sonicated electrodes were immersed in methanol containing 18.75 mM tyramine, 6.25 mM 3-(4-hydroxyphenyl) propionic acid, and also 0.3 M NaOH. After electropolymerization, the POPA-modified gold electrodes were washed with water and dried under an argon flow. Impedance spectroscopy using electrodes with POPA showed a decrease in C_dl_ and R_ct_ to approximately half of the corresponding values for polytyramine, which reduces losses. Biotinylated PrPC was linked to the POPA copolymer also through a biotin/neutravidin bridge. This made it possible to attach biotinylated PrPC with high affinity (K_d_~10^−15^ M) as a bioreceptor. The developed biosensor showed specificity for the detection of synthetic AβO, demonstrating a linear response.

**Figure 9:**
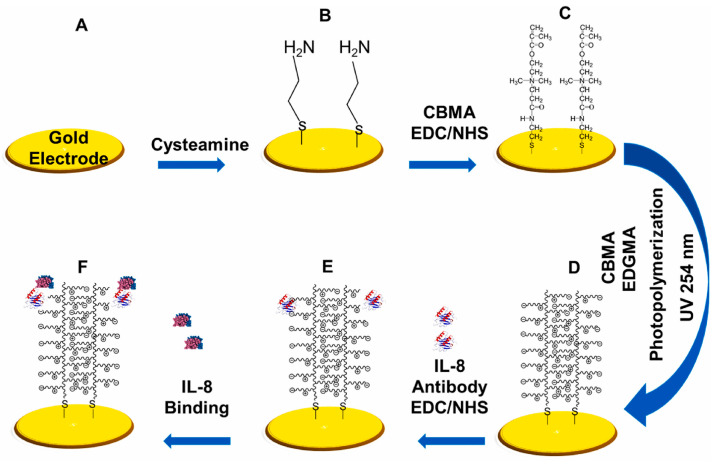
Schematic presentation of manufacturing steps of a biosensor with a Au electrode: (**A**)—Au electrode without functionalization; (**B**)—adsorption of cysteamine on the gold surface; (**C**) covalent binding of carboxybetaine monomer (CBMA) in the presence of EDC/NHS for activation; (**D**)—photopolymerization of the CBMA monomer in solution led to the formation of a zwitterionic polymer pCBMA with a CBMA-receptive interface for covalent binding of the IL-8 polyclonal antibody (Ab) in the presence of EDC/NHS (**E**) to capture the target analyte (**F**). Reprinted from [102] with permission of Elsevier provided by Copyright Clearance Center.

Hassan-Nixon et al. [102] presented a label-free affinity impedimetric biosensor using the zwitterionic polymer pCBMA for the quantitative detection of interleukin-8 (IL-8) present in nasal epithelial fluid (NELF). The entire sensor formation procedure is presented in Figure 9. The biosensor showed a wide detection range of the target protein, ranging from 55 fM to 55 nM with an LOD of 10 fM. The biosensor showed high selectivity for IL-8 and 90% reproducibility when using untreated NELF samples. The time to obtain the result is 15 min.

To provide a wide detection range of a polymer-based biosensor, nanocomposites are synthesized using nanomaterials [111,114,118,119]. Thus, Aydın et al. [118] presented a simple and reliable immunosensor for detecting the smallest change in cytokeratin fragment subunit 19 (CYFRA 21-1), a biomarker for lung cancer. The proposed immunosensor was fabricated using a C45-PTNH2 (carbon black C45/polythiophene containing amino-terminal groups) nanocomposite, which has excellent biocompatibility, is low cost, and has an electrically conductive surface. The biorecognition molecules of anti-CYFRA 21-1 were attached to the electrode utilizing the amino-thermal groups of the PTNH_2_ polymer via a relatively simple procedure. The concentration range for R_ct_ was 0.03 pg/mL to 90 pg/mL. The LOD and LOQ of the proposed system were 4.7 fg/mL and 14.1 fg/mL, respectively.

Biopolymers can also be used in the development of impedimetric biosensors to modify the working electrode. In [120], electrodes based on graphite paper were modified with polyglutamic acid using electropolymerization and activated in an EDC/NHS solution to create a leptin biosensor. Xia et al. [121] used biotinylated phenylalanine (amino acid) monomers to modify the electrode and self-assemble into various nanostructures by controlling the pH of the solution. In particular, the strong interaction between biotin and streptavidin (SA) linker on the electrode surface initiated the formation of networks. The method was successfully used to monitor caspase-3 activity in HeLa cells treated with various anticancer reagents. In [117], to create a flexible, fully organic, and biodegradable impedimetric biosensor, the electrode was formed using a biocomposite based on photoreactive silk sericin combined with a conductive polymer. Functional electrodes were printed on flexible fibrion substrates. The biosensor was developed for the analysis of vascular endothelial growth factor (VEGF) by immobilizing anti-VEGF on a conductive matrix. In [122], to detect the S1 protein of the SARS-CoV-2 virus spike, a carbon electrode was modified with gelatin.

In addition to antibodies, other types of biorecognition elements, such as aptamers, peptides, molecular imprinted polymers (MIPs), etc., are used. They can be synthesized in the presence of a template (analyte) and are a promising alternative to biomolecules in the design of biosensors [119]. Since the principle of creating biosensors based on MIPs differs from the immobilization of working electrodes based on other materials, including polymers [113,119,123,124,125,126,127], in this section, we highlight MIPs as a material for electrode modification.

As an example of the use of MIPs to create an impedimetric biosensor, let us consider a biosensor for determining Tau protein (p-Tau-441), a biomarker of Alzheimer’s disease [123]. A MIP was deposited on carbon SPE by electropolymerization of 3-aminophenol (AMP) in the presence of a protein matrix (p-Tau). After washing the resulting AMP polymer with p-Tau, incubation was carried out in a solution of proteinase K to cleave p-Tau (for 2.5 h). After protein removal and washing, the electrochemical stabilization step was carried out. The detection limit of p-Tau-441 for MIP-based sensors was 0.02 pM in PBS buffer pH 5.6. The linear response range of the biosensor was between 2.18 pM and 2.18 nM.

**Figure 10:**
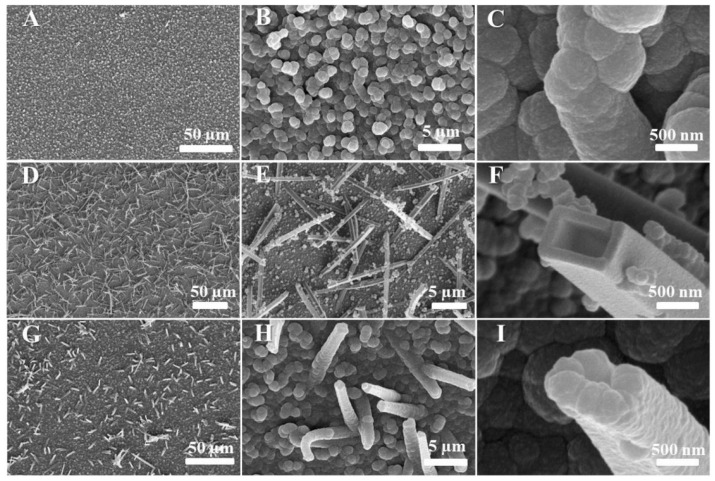
FE-SEM images of electropolymerized coatings of FTO/PPy (**A**–**C**), FTO/PPy-MO NIP (**D**–**F**), and FTO/PPy-MO DMIP electrodes (**G**–**I**). The DMIP electrode was prepared by imprinting 100 ng/mL of each biomarker AFP and CEA. Reprinted from [113] with permission of Elsevier provided by Copyright Clearance Center.

In [113], a dual-template molecular imprinted polymer (DMIP) biosensor was fabricated by electropolymerization to detect carcinoembryonic antigen (CEA) and alpha-fetoprotein (AFP) as biomarkers of lung cancer. The protein matrices (CEA and AFP antigens) were removed by washing in sodium hydroxide solution. The polymer used was polypyrrole (PPy) with the addition of methyl orange (MO), which increased the conductivity of PPy and caused the formation of rectangular-shaped nanotubes (PPy-MO), as shown in Figure 10. The LOD values of 1.6 pg/mL and 3.3 pg/mL were obtained for CEA and AFP, respectively. It has been shown that the self-assembly of PPy-MO nanotubes made it possible to increase the specific surface area of the biosensor and thus further expand the response range.

A specific member of the synthetic organic compound polymers should be mentioned in this section. Thus, in [128], to create a biosensor for D-dimer, the carbon electrode was modified with negatively charged synthetic phospholipid-dihexadecyl phosphate and gold nanoparticles. 

**Figure 11:**
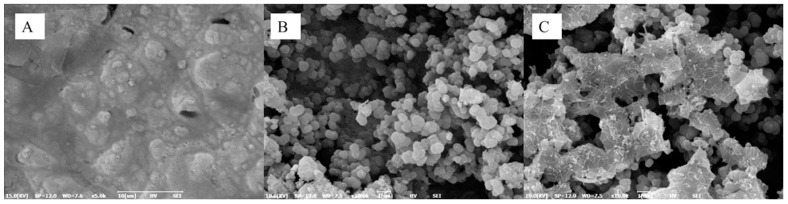
SEM images of electrode modifications AuE (**A**), AuE/4-ATP/fullerene (**B**), and AuE/4-ATP/fullerene/PAMAM (**C**). Reprinted from [131] with permission of Elsevier provided by Copyright Clearance Center.

In [129,130], dendrimers were used to modify electrodes and for subsequent immobilization. For example, Erdem et al. [129] presented an aptasensor based on a graphite electrode coated with a polyamidoamine dendrimer (PAMAM) with 16 carboxyl groups on the surface (PAMAM G2). A DNA aptamer selective for activated protein C (APC) was immobilized onto the dendrimer. The sensitivity of the system was 0.74 μg/mL (0.46 pmol in 35 μL of sample) in buffer solution and 2.03 μg/mL (1.27 pmol in 35 μL of sample) in serum. In [131], fifth-generation polyamidoamine dendrimers PAMAM (G5) were used as part of a nanocomposite, which was used to detect Fetuin-A (HFA) in real blood samples. To manufacture the biosensor, a coating was formed on the surface of the gold electrode by sequentially applying a self-assembly monolayer (SAM) of 4-aminothiophenol (4-ATP), fullerene, and PAMAM-NH_2_ (G5, generation 5). As can be seen from Figure 11, when fullerenes are deposited, a large increase in the specific surface area of the electrode is observed. Before applying polyhydroxyl fullerenes (C_120_O_30_(OH)_30_), a fullerene solution was incubated in HEPES buffer with the addition of EDC and NHS to activate OH groups. To obtain the final structure, a solution of fifth-generation polyamidoamine dendrimers PAMAM(G5) in phosphate buffer pH = 6.8 was applied to the AuE/4-ATP/fullerene electrode and incubated for 1 h. The activation of amino groups of AuE/4-ATP/fullerene/PAMAM was carried out in a glutaraldehyde solution. Anti-Fetuin-A antibodies were incubated for 2 h. Open groups of glutaraldehyde were blocked with the amino acid glycine. The LOD and LOQ of the biosensor were calculated as 0.48 ng/mL and 1.46 ng/mL, respectively.

Examples of impedimetric biosensors using polymeric materials are given in Table 4.

### 3.5. Metal Complexes of Porphyrins, Phthalocyanines, and Metal–Organic Frameworks

In addition to polymers, dendrimers, and SAMs, we can highlight the use of metal–organic frameworks (MOFs) [132,133,134,135], as well as metal complexes of porphyrins [136] and phthalocyanines [137,138].

In [136], an impedimetric biosensor based on a zirconium porphyrin complex was developed for the detection of neuron-specific enolase (NSE). A complex based on Zr(III) and tetra(4-aminophenyl)porphyrin (Zr-TAPP) provided a strong affinity for the antibody (anti-NSE). To create the biosensor, the Zr-TAPP dispersion was deposited on a Au electrode (AE) and dried. The resulting Zr-TAPP/AE was incubated in an anti-NSE solution at 4 °C for 40 min. Anti-NSE/Zr-TAPP/AE was incubated with an NSE solution for further electrochemical measurements. A porphyrin-based Zr-MOF immunosensor (PCN-224) was similarly constructed. The developed biosensor demonstrated an NSE LOD value of 7.1 fg/mL and a linear range from 10.0 fg/mL to 2.0 ng/mL.

Centane et al. [137] developed a biosensor for human epidermal growth factor receptor 2 (HER2) and studied the use of cobalt tetraphenylacetic acid phthalocyanine and cobalt tetraphenylpropionic acid phthalocyanine, which, after activation in DCC/NHS, were used as a platform to immobilize the HB5 aptamer. Also, to increase the specific area of the sensor, phthalocyanines were associated with nanoparticles based on cerium oxide. The aptasensors showed good LOD values for HER2 (all less than 0.2 ng/mL) with high stability. 

**Figure 12:**
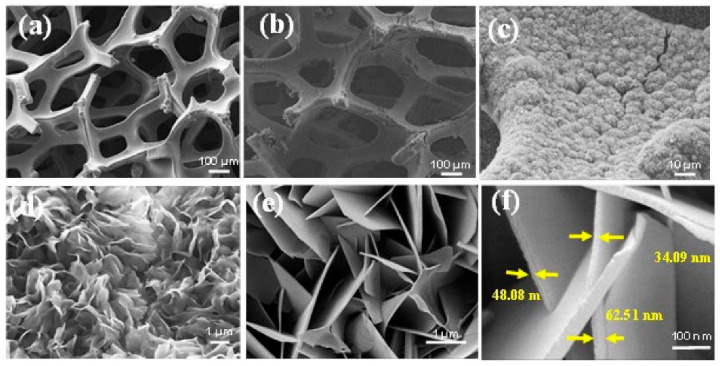
The original nickel foam and the resulting FeCo-MOF/NF: (**a**) SEM images of bare porous NF, (**b**–**e**) FeCo-MOF grown on the NF skeleton (FeCo-MOF/NF) after in situ hydrothermal reactions using precursors, ligands, and Ni foam skeleton at different magnifications. The marks in (**f**) are the average thickness of FeCo-MOF nanosheets grown on porous NF substrates. Reprinted from [134] with permission of Elsevier provided by Copyright Clearance Center.

Vijayaraghavan et al. [134] developed an impedimetric immunosensor based on bimetallic amine functionalized FeCo metal–organic frameworks uniformly grown on porous solid nickel substrates (FeCo-MOF/NF) as a transducer for the detection of oral squamous cell carcinoma (OSCC). The functional group providing selectivity and rapid sensitivity to the target analyte is IL-1RA. The antibody-immobilized FeCo-MOF/NF electrode was the working electrode. To fabricate the sensor, commercial nickel foam (NF) was cut into 3 × 3 cm pieces. The growth of FeCo-MOF nanoarrays on a porous nanofilter (denoted as FeCo-MOF/NF) was performed using a hydrothermal reaction. The original nickel foam and the resulting FeCo-MOF/NF are shown in Figure 12. The immunosensor showed a wide linear dynamic range for IL-1RA detection (10 fg/mL to 10 ng/mL) with an LOD value of 7.30 fg/mL in buffer and 7.22 fg/mL in serum conditions and an LOQ value of 22.14 fg/mL in PBS and 21.88 fg/mL in serum.

Gupta et al. [133] used Cu_3_(BTC)_2_-based metal–organic frameworks (MOFs) embedded in a conductive polymer (PANI) as a coating on the electrode surface. This composite was used to develop an impedance sensor for the cardiac biomarker troponin I (cTnI). An electrically conductive composite thin layer was coupled with anti-cTnI antibodies. Cu_3_(BTC)_2_ (where BTC = benzene-1,3,5-tricarboxylate) was synthesized at room temperature from H_3_BTC (benzene-1,3,5-tricarboxylic acid) and Cu(NO_3_)_2_ using the solvothermal method at room temperature as a solution in distilled water, ethanol, and dimethylformamide with the addition of triethylamine. The resulting immunosensor made it possible to selectively register cTnI in a clinically significant concentration range from 1 to 400 ng/mL. The cTnI analysis time was 5 min.

Examples of impedimetric biosensors using metal complexes of porphyrins, phthalocyanines, and metal–organic frameworks are given in Table 5.

### 3.6. Metal Oxides and Dielectrics

Metal oxides (MO) and nanomaterials based on MO are often used as substances for the functionalization of surfaces and subsequent functionalization. Metal oxides are typically highly electrically resistive semiconductors and are primarily used for biosensors operating in the non-faradaic EIS mode, in which the main contribution to the impedance spectrum is the capacitance change. Nanomaterials made of metal oxides, like other nanomaterials, provide a high specific surface area for immobilization. Among metal oxides, nanoparticles and nanostructures of TiO_2_, ZnO, WO_3_, and others are most often used [51,139,140,141,142,143,144,145]. The charge transfer resistance R_ct_ in non-faradaic EIS biosensors can tend to infinity if dielectric materials are used for immobilization [146,147,148]. For the same purpose, electrodes can be separated from semiconductor nanomaterials by a dielectric layer, such as glass.

The work [149] presents a biosensor for biomarkers of sepsis-procalcitonin (PCT) and C-reactive protein (CRP) in human serum and blood. The biosensor is based on recording the impedance of the reaction between the target analyte and a specific probe on the surface of zinc oxide (ZnO). Interdigitated gold electrodes on a polyimide substrate were used as electrodes in the biosensor. To increase sensitivity, a thin ZnO film was deposited on gold electrodes using RF magnetron sputtering. Thiol-based crosslinker molecule dithiobis (succinimidyl propionate) (DSP) in DMSO was incubated at room temperature in the dark for 90 min for effective thiol functionalization. Each sensor was individually functionalized with a PCT- or CRP-specific capture probe. Finally, 10 μL of Superblock was added to the sensor and incubated for 15 min to hydrolyze any unbound binding sites. A frequency of 10 Hz was selected for the calibration dose response of PCT in human serum and whole blood, and a frequency of 100 Hz was selected for CRP in human serum and whole blood. The LOD values for PCT and CRP were 0.10 ng/mL and 0.10 μg/mL, respectively.

**Figure 13:**
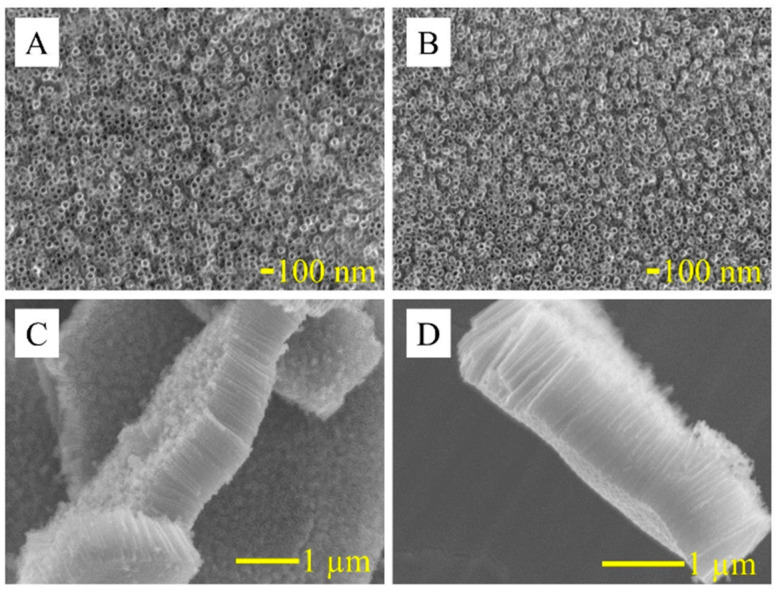
SEM images of top view (**A**,**B**) and cross-view (**C**,**D**) of TNT (**A**,**C**) and TNT annealed in argon (**B**,**D**) at 550 °C for 2 h. Reprinted from [141], license CC BY-NC-ND 4.0.

In [141], a titanium dioxide nanotube (TNT) matrix was used to create an impedimetric sensor for the detection of human interleukin 6 (IL-6), interleukin 8 (CXCL8, IL-8), and tumor necrosis factor (TNFα). Antibodies were used as recognition elements. To form TNT, titanium foil was sonicated in acetone, ethanol, and distilled water and dried in nitrogen. The formation of oxide layers of nanotubes was carried out by electrochemical anodization in solutions of ethylene glycol with NH4F. The resulting titanium dioxide nanotube array is shown in Figure 13. Antibodies were immobilized onto TNT by physical adsorption. The immunosensor demonstrated good selectivity and high sensitivity up to 5 pg/mL, which is the standard concentration of analyte proteins in human blood. The detection range for the three cytokines was 5–2500 pg/mL in 0.01 M phosphate buffer (pH 7.4).

**Figure 14:**
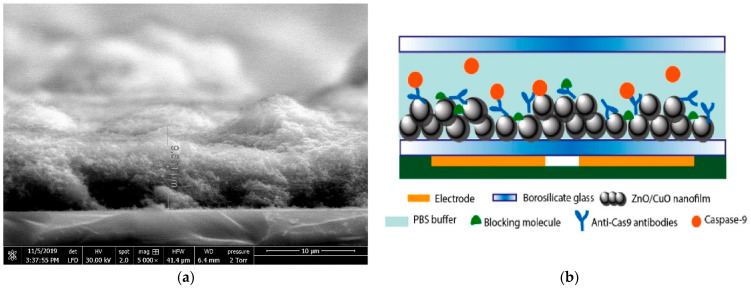
Non-faradaic impedimetric biosensor monitoring of caspase-9 in mammalian cell culture: (**a**) SEM image of a film of ZnO/CuO nanoparticles; (**b**) illustration of a cross-section of the biosensor surface and biorecognition interactions between anti-cas9 and caspase-9. Reprinted from [51], license CC BY-NC-ND 4.0.

Chen et al. [51] developed a biosensor to detect caspase-9, an enzyme that is involved in apoptosis or programmed cell death. In this work, metal oxides were used in the form of nanoparticles, and they were separated from the electrodes by a thin cover glass. To prepare the biosensor, a suspension of ZnO nanoparticles and CuO nanoparticles was applied to the cover glasses by dripping and then dried in an oven at 50 °C for 4 h. The resulting coating is shown in Figure 14a. For immobilization, an antibody solution (rabbit anti-cleaved caspase-9 IgG) was dropped onto the sensor area and incubated for 3 h at room temperature. The biosensor was positioned over a pair of coplanar electrodes formed from 35 μm copper on a printed circuit board. A cross-section of the biosensor is shown in Figure 14b. The sensor response was assessed using readings obtained at a frequency of 6 MHz. As a result, the sensor demonstrated an LOD value of about 0.07 U/mL caspase-9 concentration. The average analysis time was 45 min, including preparation. A similar biosensor structure based on ZnO/CuO nanoparticles was used in [150] to create a biosensor for C-reactive protein.

ZnO and CuO nanoparticles were also used to create a biosensor for C-reactive protein (CRP) (CRP) in [151]. However, in this work, a 3D membrane was fabricated based on nitrocellulose membranes and ZnO and CuO nanoparticles. The use of a 3D membrane aims at more efficient capturing of antibodies by monoclonal mouse anti-human C-reactive protein (4C28). Cellulose membranes were immersed in nanoparticle suspensions and treated with ultrasound to form a nanoparticle-based 3D membrane. The sonicated nano-ZnO and nano-ZnO/CuO nitrocellulose membranes showed detection limits of 27 and 16 pg/mL, respectively. The authors noted the ease of fabrication of this sensor, the lack of contact between the immobilized nanomaterial and the electrodes, and the absence of the need to use a redox active species for measurement.

In [147], immobilization was carried out directly on a glass substrate. An impedimetric biosensor was fabricated with Au electrodes for the quantification of human serum albumin (HSA). The glass sensing surface between two adjacent Au electrodes was modified with 3-aminopropyltriethoxysilane (APTES) to silanize and subsequently bind to anti-human serum albumin (AHSA). Before APTES modification, the samples were cleaned and treated with a solution of H_2_SO_4_ and H_2_O_2_ (1:3) to functionalize the surface with hydroxyl groups. The HSA LOD value limit of the developed biosensor was about 2 × 10^–4^ mg/mL. 

In [146], a biosensor for detecting HSA was fabricated based on a dielectric, silicon nitride Si_3_N_4_, and anti-HAS recognition agent. In general, the electrode had a Si-p/SiO_2_/Si_3_N_4_ structure. The Si_3_N_4_ sample was similarly treated with a Piranha solution of H_2_SO_4_ and H_2_O_2_ (1:3) for modification with reactive OH groups. Silanization was carried out using triethoxysilane aldehyde (TEA) in the vapor phase. The detection limit of HSA was 10^–14^ M. 

Examples of impedimetric biosensors implemented using metal oxides and dielectrics are given in Table 6.

### 3.7. Other Nanomaterials

This section describes nanomaterials that are somewhat less common but are also successfully used to create impedimetric biosensors. Such materials include colloidal quantum dots [152,153], which during the synthesis process are formed with a shell of ligand molecules, two-dimensional nanomaterials from other semiconductor compounds [154,155,156], and, finally, magnetic nanoparticles [157,158].

**Figure 15:**
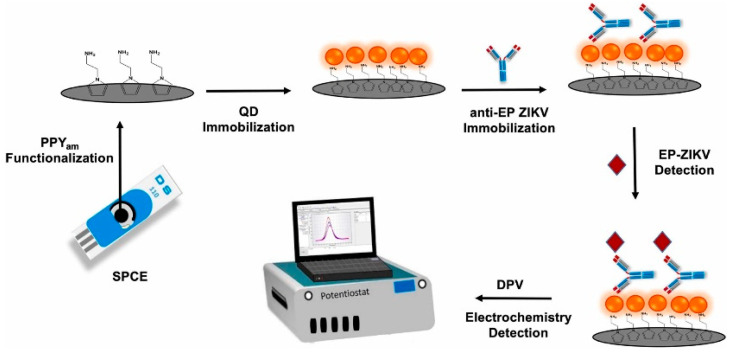
Schematic representation of the stages of immunosensor formation with QD for detection of EP-ZIKV. Reprinted from [153] with permission of Elsevier provided by Copyright Clearance Center.

In [153], a biosensor for detecting Zika virus envelope protein (EP-ZIKV) was developed based on an electrode with carboxylated CdTe QDs. Quantum dots were obtained in the form of an aqueous colloidal dispersion using mercaptosuccinic acid as a stabilizing agent (ligand molecules). The size of CdTe QDs was estimated to be 3.3 ± 0.4 nm. CdTe QDs were deposited on a polymer-coated electrode. A film of polymer with amine groups 2-(1Hpyrrol-1-yl)ethanamine (Py_am_) was deposited onto the electrode electrochemically. QDs were immobilized on the surface of Pyam in the presence of EDC/NHS to promote covalent bonding between the carboxyl groups of the QDs and the amine groups of the PPy_am_ polymer film. Anti-EP ZIKV antibodies were immobilized on QDs through carboxyl groups activated in the previous step. The general preparation scheme of the electrochemical immunosensor is shown in Figure 15. It is reported that Anti-EP ZIKV antibodies were effectively immobilized on the surface of PPy_am_/QD even after 2 months of electrode storage. The biosensor had high sensitivity with an LOD value of 0.1 ng/mL and demonstrated good specificity.

**Figure 16:**
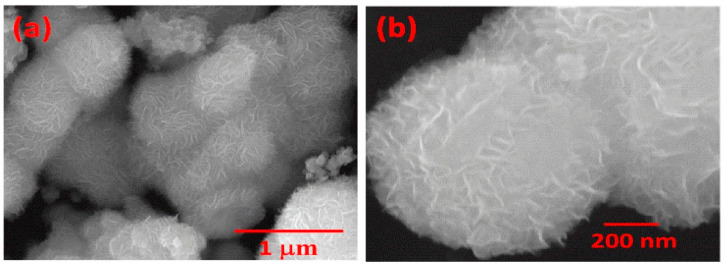
SEM electron microscopy images of MoS_2_ nanoflowers: (**a**) flower like morphology; (**b**) single bud with a more magnified petals. Reprinted from [154] with permission of Elsevier provided by Copyright Clearance Center.

A 2D semiconductor material was used to create a biosensor in which MoS_2_ nanoflower (nf) was used to create a substrate surface for direct immobilization of antibodies for binding tumor necrosis factor-α (TNF-α) [154]. The synthesis of MoS_2_ nfs was carried out by a one-step hydrothermal method for 24 h. Then, MoS_2_ was washed with a mixture of ethanol and water to separate impurities, dried at 60 °C for 5 h to obtain a black powder, and ground to obtain a homogeneous powder. The morphology of the resulting MoS_2_ was determined from electron microscopy (Figure 16). Then, a colloidal solution of MoS_2_ nfs was prepared in acetonitrile under ultrasonication. And a hydrolyzed electrode was deposited on the ITO at a potential of 50 V using a platinum plate as the second electrode. As a result, a uniform layer of MoS_2_ nfs was formed. Anti-TNF-α antibodies were then immobilized on the electrodes by incubation for 6 h. The morphology of the nanoflowers facilitated the immobilization of antibodies through physical adsorption without the use of linkers. The BSA/anti-TNF-α/MoS_2_ nf/ITO immunoelectrode demonstrated the value of LOD of 0.202 pg/mL and a linear detection range of TNF-α of 1–200 pg/mL with fairly high selectivity.

Mi et al. [155] used a hybrid of AuNPs and Ti_3_C_2_-MXenes to fabricate aptasensors for cTnI and Myo. Before fabricating the biosensor, the ITO electrode was amino-functionalized using APTMS, and a hybrid suspension was synthesized. To fabricate the biosensor, a hybrid AuNP/Ti_3_C_2_-MXene suspension was applied to the surface of an APTMS/ITO electrode and left for 30 min at room temperature, followed by washing and drying in an N_2_ stream. The AuNP/Ti_3_C_2_-MXene nanohybrid was anchored on APTMS/ITO via a Au–N bond. Next, the electrode was incubated in the aptamer solution. For the cTnI aptasensor, blocking of unbound regions was performed using MCH. For the aptasensor to Myo, blocking of unbound regions was performed with BSA. AuNP/Ti_3_C_2_-MXene captured a cTnI-specific, thiol-functionalized DNA aptamer via Au-S self-assembly, and the Myo-aptamer was bound via adsorption and metal–halate interaction between phosphate groups and titanium. The developed aptasensors demonstrated specificity and good sensitivity. The LOD for cTnI was 0.14 fg/mL and for Myo was 0.2 ng/mL.

**Figure 17:**
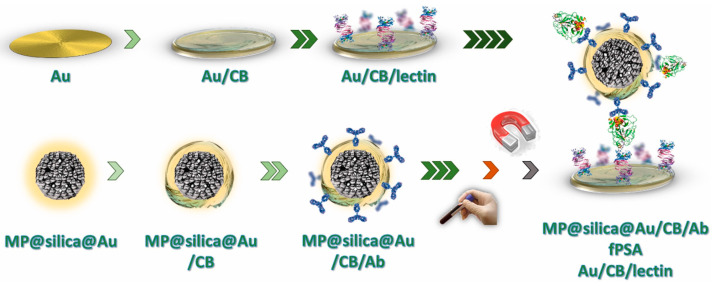
Modification stages of Au electrode using aryldiazonium (CB) carboxybetaine derivative and lectin (SNA-I). Reprinted from [157] with permission of Elsevier provided by Copyright Clearance Center.

In [157], particles with a magnetic core and a gold shell were used to create a biosensor for PSA. Ferrite cores Mn_0.61_Zn_0.42_Fe_1.97_O_4_ were synthesized by hydrothermal method and then encapsulated into silica. The Au nanoshell was deposited using the seed-and-growth method. MP@silica@Au and the Au electrode surface were modified using carboxybetaine aryldiazonium derivative (CB) to obtain the SAM layer. For clarity, all stages are presented in Figure 17. At the first stage, a SAM layer carrying carboxybetaine was formed using cyclic voltammetry, which subsequently served for covalent immobilization of the lectin. The bottom row shows the MP@silica@Au composite spontaneously modified with a CB derivative and then with an antibody (Ab, anti-fPSA, chemically treated). After fPSA enrichment from a human sample using MP@silica@Au/CB/Ab, a sandwich was prepared using a permanent magnet, and the signal was assessed electrochemically. The developed biosensor demonstrated the ability to detect PSA down to 1.2 fM.

Examples of impedimetric biosensors using other nanomaterials and dielectrics are given in Table 7.

## 4. Biorecognition Elements for Protein Detection

Since impedimetric biosensors respond to the binding of an analyte to a biorecognition element (or ligand), these recognition elements must have the following set of qualities: (i) high affinity and specificity for the target protein to ensure reliable and sensitive detection, (ii) high stability for maintaining activity and functionality over long periods of time and under different operating conditions, (iii) simplicity and efficiency of immobilization, that is, the ability to be easily and effectively attached to the electrode surface with minimal impact on their activity and functionality to ensure optimal exposure of binding sites and minimization of undesirable side reactions, and (iv) low cost for mass production with low costs of materials and technology.

There are several classes of recognition elements that can be used for impedimetric detection of proteins, such as antibodies, aptamers, peptides, enzymes, affimers, affibodies, nanobodies, and molecularly imprinted polymers [159,160,161,162,163,164,165]. The binding of the analyte to the recognition element leads to a change in the electrical properties on the electrode surface. These changes may be due to various factors, including the following:

  (i). Change in thickness and dielectric constant of the recognition element layer. The binding of the analyte to the recognition element increases the thickness and dielectric constant of the layer, which leads to a change in capacitance [166] and an increase in resistance at the electrode surface [167]. This effect is observed when using thin layers of recognition elements such as antibodies or aptamers [168,169].
 (ii). Change in charge and conductivity of the recognition element layer. The binding of the analyte to the recognition element can change the charge and conductivity of the layer, which leads to a change in the potential and current at the electrode surface [170]. This effect is observed when using charged recognition elements such as nucleic acids [171] or peptides [172].
(iii). Change in the conformation of the recognition element. The binding of an analyte to a recognition element can cause a change in its conformation, which leads to a change in capacitance and resistance to direct current on the surface of the electrode [173,174]. This effect is observed when using recognition elements that can change their structure when binding to the analyte, such as aptamers or enzymes [29,175,176].

Biorecognition elements for impedimetric biosensors can be classified according to various criteria, including the following:

 (i). Origin. Recognition elements can be natural or synthetic [177]. Natural recognition elements such as antibodies or enzymes usually have higher specificity and selectivity but are also more expensive, difficult to obtain and store, and are subject to degradation [178]. Synthetic recognition elements, such as aptamers, peptides, and nanobodies, generally have a lower cost, greater ease of synthesis and modification, and higher stability and regenerability [179].
(ii). Size and molecular weight. Recognition elements can have different sizes, from several nanometers to several hundred nanometers, and different molecular weights. The size of the recognition element affects its properties, such as affinity, binding kinetics, mass transfer, electron transfer, and signal-to-noise ratio [180,181]. In general, the smaller size and molecular weight of the biorecognition element lead to faster and more sensitive detection due to the greater number of binding sites immobilized on the electrode surface [182]. In addition, the larger size and molecular weight of the biorecognition element increases its steric specificity, thereby reducing the likelihood of unwanted interactions with other molecules. This is especially important when studying the binding abilities of peptides and aptamers.

When choosing a biorecognition element and its characterization, an important parameter is the method of its immobilization. Recognition elements can be immobilized on the electrode surface in various ways, such as physical adsorption, covalent binding, biotin–avidin interaction, electropolymerization, self-assembled monolayers, etc. [33,183,184,185,186]. The first two methods are the most common. During physical adsorption, biorecognition agents are attached to the surface of the transducer due to physical forces, such as van der Waals forces, hydrophobic interactions, electrostatic forces, etc. [187]. With covalent binding, biorecognition elements are attached to the surface of the transducer by forming covalent bonds with the surface of the electrodes. This method provides high stability, regeneration ability, and orientation of biorecognition elements but may require complex and expensive procedures, such as surface activation, selection of reagents, and control of reaction conditions [188]. Biotin–avidin interaction is a method in which a ligand is attached to the surface of a transducer using biotin and avidin (or streptavidin) [189]. Biotin is a small molecule that can be synthetically attached to a ligand, and avidin (or streptavidin) is a protein that has a high affinity for biotin. This method provides high specificity, stability, and regenerability of the biorecognition element but requires additional steps, such as biotinylation of the biorecognition element, immobilization of avidin (or streptavidin) on the surface of the transducer, etc. [190]. Electropolymerization is the electrochemical deposition of a polymer film material on the surface of an electrode with the simultaneous inclusion of biorecognition elements in the polymer matrix [191,192]. This method has advantages such as simplicity, stability, selectivity, and the ability to control the properties of the polymer film. However, this method also has disadvantages, such as a limited selection of biorecognition elements and a possible decrease in their activity [193]. Monolayer self-assembly is a method in which a ligand is attached to the surface of a transducer using molecules that spontaneously form an ordered monomolecular layer on the surface [194,195]. These molecules have two functional groups: one group binds to the surface of the transducer, and the other group binds to the biorecognition element. This method provides high orientation, stability, and regenerability of the ligand but requires careful selection of SAM molecules, control of concentration, temperature, time, etc. [196].

The immobilization method affects the density, orientation, activity, and stability of the recognition element, as well as the electrical properties of the electrode [184]. In general, covalent binding provides higher density, stability, and reproducibility of biorecognition elements on electrodes but also lower activity and electron transfer than physical adsorption [185,186]. Therefore, the choice of ligand immobilization method depends on the specific goals and conditions of the analysis, as well as on the type of biorecognition element and transducer. In some cases, it may be advisable to use a combination of different immobilization techniques to achieve optimal results. For example, one can use physical sorption to create a first ligand layer and then chemical sorption to create a second ligand layer of a different type or orientation.

Next, we will consider the features of using various biorecognition elements for detecting proteins using impedimetric biosensors.

### 4.1. Impedimetric Detection of Proteins Using Antibodies

One of the most common biorecognition elements used in biosensors are antibodies. Antibodies are glycoproteins that are produced by the immune system in response to foreign agents (antigens) such as bacteria, viruses, toxins, and others [197,198,199]. Therefore, biosensors that use antibodies as a recognizer are often called immunosensors [200,201]. Antibodies have a Y-shaped structure of four polypeptide chains that are able to bind a specific antigen and form an antibody–antigen complex. Antibodies are capable of recognizing and binding to specific epitopes on the surface of an antigen with high specificity and affinity. Depending on how many epitopes of a single-antigen molecule they can recognize, antibodies can be either mono- or polyclonal [202]. Antibodies are widely used in immunosensors because they can detect a variety of protein analytes with high sensitivity and selectivity. However, antibodies have some disadvantages, such as high synthesis cost, difficulty in production, low stability, and the possibility of aggregation, degradation, and denaturation when immobilized on the electrode [203].

When creating impedimetric immunosensors, it is important to prevent possible nonspecific binding of the analyte to nontarget components, which introduces a spurious signal into the impedance measurements. For this purpose, free spaces on the surface of the chip that were not functionalized with antibodies are blocked with BSA [204], casein [205], twin-surfactants [206], commercial solutions such as SmartBlock™ [207], and other compounds.

The efficiency of antibody binding with the target protein may depend on the amount of immobilized ligands on the surface of the electrodes [208,209]. It was noted in [78] that excess antibody molecules to the Aβ42 protein bound on the surface created a steric barrier for the protein molecules to approach the antibody molecules in the correct orientation. Therefore, to achieve the required sensitivity, the authors had to use a fairly long incubation period of about 60 min.

**Figure 18:**
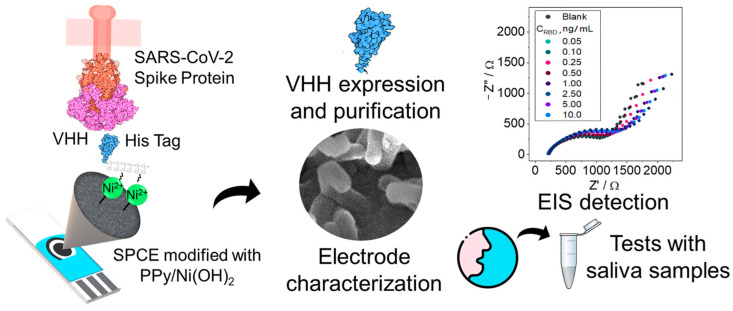
COVID-19 impedimetric biosensor based on polypyrrole nanotubes, nickel hydroxide, and VHH antibody fragment. Reprinted from [210] with permission of Elsevier provided by Copyright Clearance Center.

Another important property of immobilized ligands that determines the efficiency of binding is their uniform orientation on the surface of the electrodes. Santos et al. [210] proposed a structure based on polypyrrole nanotubes (Figure 18) linked via Ni(OH)_2_ ligation to an engineered antibody heavy chain antigen-binding (VHH) moiety called Sb#15. This fragment is capable of selectively binding the receptor (RBD) of SARS-CoV-2. The developed structure allows the correct orientation of antibody immobilization for sensitive detection of the SARS-CoV-2 antigen. The position of the His tag on a protein can be controlled by genetic engineering, resulting in the uniform orientation of the antibody during immobilization. The developed sensor requires a small volume of saliva for analysis and provides results in 15 min without additional sample preparation with a sensitivity of up to 0.01 pg/mL. In [114], to detect the specific spike receptor protein (RBD), a structure based on a thiophene monomer with a substituted epoxy functional group was fabricated. It was electrodeposited onto a disposable indium tin oxide (ITO) platform in the presence of acetylene black. The resulting composite provided the correct attachment points for antibody binding and also supported the biosensor structure. The detection limit was 0.58 fg/mL. Adesina et al. [211] used a self-assembled monolayer (SAM) of 4-mercaptophenylboronic acid (4-MPBA) to orient immobilized anti-C-reactive protein (CRP) antibodies. Controlling ligand orientation was key to eliminating false positives and negatives during sample analysis. The resulting sensor made it possible to achieve a detection limit of 0.10 μg/mL.

One of the common problems of immunosensors is the storage stability of manufactured structures with immobilized antibodies [212,213,214]. Manufacturers and researchers are trying to increase it by creating new biosensor structures. Zhang et al. [215] presented a biosensor for the detection of apolipoprotein-A1 (Apo-A1), a biomarker for bladder cancer. The sensor is based on a glassy carbon electrode (GCE) coated with molybdenum and graphene nanoparticles on which antibodies to Apo-A1 are attached. The sensor also uses other lead and gold nanoparticles that are anchored with other Apo-A1 antibodies. When these antibodies bind to Apo-A1, they form a sandwich structure that changes the resistance of the electrode when an electrical current is passed through. The sensor showed a good dependence of resistance on Apo-A1 concentration in the range from 1 pg/mL to 1 μg/mL. To check the stability of the biosensor, the relative change in impedance %ΔR_et_ was regularly measured. At a storage temperature of 4 °C, %ΔR_et_ remained almost unchanged for a month and then gradually decreased and remained at the level of 90.1% after 2 months. The work [216] considers an immunosensor for detecting brain natriuretic peptide (BNP), which is a biomarker of cardiovascular diseases. The sensor consists of a graphite electrode coated with gold nanoparticles and graphene with chitosan, to which antibodies to BNP are covalently attached. The sensor showed high sensitivity and specificity for BNP in the range from 0.01 to 1000 pg/mL. The sensor was consistent with the commercial method and retained its activity for 60 days when stored under low-temperature conditions. The sensor did not respond to other proteins such as BSA or IgG. The sensor could be restored using an alkaline solution or pyranic acid.

Lee et al. [167] described the development of a sensor to detect ubiquitin C-terminal hydrolase L1 (UCH-L1), which is a biomarker of traumatic brain injury. The sensor is based on the use of antibodies immobilized on a self-assembled monolayer (SAM), which can be lyophilized and frozen with added sugar for long-term storage. The sensor has a disposable screen-printed electrode, which increases measurement reproducibility. The sensor operates on the principle of impedance spectroscopy with one optimal frequency, which simplifies the system and reduces measurement time to 5 min. The sensor has a low LOD value of 1 pM and a wide dynamic range from 1 pM to 10 pM, which makes it possible to distinguish between normal and elevated levels of UCH-L1 in the blood. The sensor does not require the separation of bound and free antibodies or washing. In artificial serum, the sensor is less sensitive due to the high salt concentration, which affects the solution resistance and the flow of the redox probe (ferricyanide). To overcome this problem, it is necessary to improve the reproducibility of sensor manufacturing.

### 4.2. Aptamer-Based Impedimetric Biosensors

Another common group of ligands for impedimetric biosensors is the aptamers. Aptamers are short, single-stranded nucleic acids (DNA or RNA) that can bind to a variety of molecular targets, such as proteins, peptides, amino acids, nucleotides, ions, and small molecules, with high specificity and affinity [26,217]. Aptamers are obtained using the systematic evolution of ligands with the exponential enrichment (SELEX) method and methods based on it [218,219]. Aptamers can also change their conformation upon binding to an analyte, which can lead to a change in electrical impedance at the electrode surface. Impedimetric aptasensors are fairly simple and fast diagnostic devices that do not require the use of labels and lengthy sample preparation [220].

Aptamers have a number of advantages over antibodies, such as low cost, chemical synthesizability, high thermal stability, and the possibility of modification and regeneration. The authors of [25] conducted a comparative study of two types of bioreceptors—an aptamer and an antibody—to detect the cancer marker HER2 on a glass–carbon electrode. The electrode is modified with graphene quantum dots with gold nanoparticles and a porphyrin binuclear framework. The aptamer and antibody are attached to them via an amide bond. Aptasensors showed better detection limit values than immunosensors. The developed aptasensor is also superior to the immunosensor in terms of repeatability and storage, while the immunosensor showed poor regeneration capabilities. Ramanathan et al. [221] presented a biosensor for diagnosing SARS-CoV-2 using aptasensing based on a gold interdigitated electrode (AuIDE) with nanodiamonds. The aptasensor can detect the nucleocapsid protein (NCP) of SARS-CoV-2 with high sensitivity and specificity. The detection limit of the aptasensor is 0.389 fM, and the linear range is from 1 fM to 100 pM.

**Figure 19:**
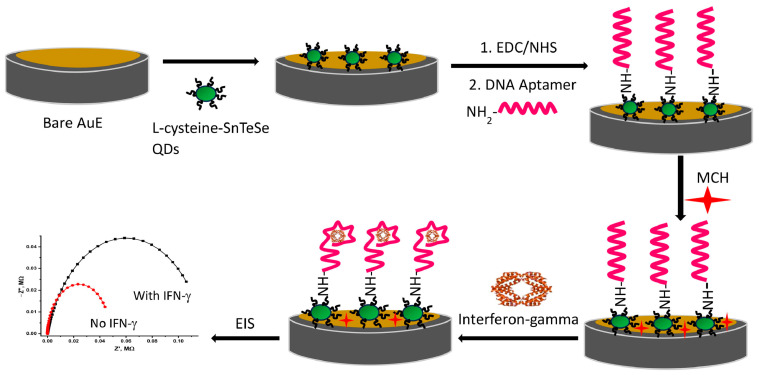
Preparation of L-cysteine-SnTeSe QD aptasensor. Reprinted from [152] with permission of Elsevier provided by Copyright Clearance Center.

Chemical modifications can be used to improve the sensitivity of aptasensors and improve the immobilization of ligands on the electrode surface. Januarie et al. [152] proposed an aptasensor for the detection of interferon-gamma, a biomarker of tuberculosis, based on SnTeSe metal dichalcogenide quantum dots (Figure 19). The surface of the quantum dots is modified with short chain L-cysteine peptides to improve stability and solubility. The LOD for the developed sensor was 0.151 pg/mL, and the response time was about 8 min. The aptasensor also had good selectivity for interferon-gamma in the presence of interference. When evaluated on a clinical sample, the aptasensor showed a good recovery range of 98–105%, indicating its suitability for infectious disease monitoring.

The work [222] describes a method for determining the protein marker of breast cancer MUC1 using an electrode coated with nanofibers, nanotubes, and gold nanoparticles with an attached aptamer that binds to MUC1. The method can detect MUC1 in the range from 5 to 115 nM with a detection limit of 2.7 nM. Aptamers are thermally and chemically stable and remain active after several cycles of denaturation/renaturation. The stability of the modified electrode was tested after storage in the refrigerator for 4 and 8 weeks. The selectivity of the aptamer has been confirmed in the presence of other biological molecules such as has, PSA, urea, glucose, and HIgG.

Thus, to create new effective impedimetric aptasensors, it is necessary to design new modified electrodes using nanotechnology and chemical modification. It is also necessary to develop new methods for selecting aptamers sequences.

### 4.3. Peptide “Aptamers”

Peptides are short chains of amino acids that can have specificity and affinity for certain proteins. Peptides can be obtained using chemical or biological synthesis, as well as using combinatorial chemistry or phage display [223,224]. Peptides can be modified with various functional groups such as thiols, amines, carboxyls, biotin, streptavidin, etc., as well as various electroactive labels [225] to ensure specific and stable binding to the electrode [226]. Peptides can be targeted on the electrode surface using various strategies, such as the use of spatial probes, multivalent ligands, selective receptors, etc., to ensure optimal exposure of binding sites [227].

Peptides have a number of advantages over more widely used antibodies and aptamers, such as low cost, ease of synthesis and modification, high stability and regenerability, low immunogenicity and toxicity, and the ability to be designed as needed [228]. However, peptides also have a number of disadvantages, such as low affinity and specificity compared to antibodies, low catalytic activity compared to enzymes, and the potential for denaturation, aggregation, and degradation under certain conditions. However, the advantages of peptides often outweigh their disadvantages, especially when compared to more complex immunoassays.

**Figure 20:**
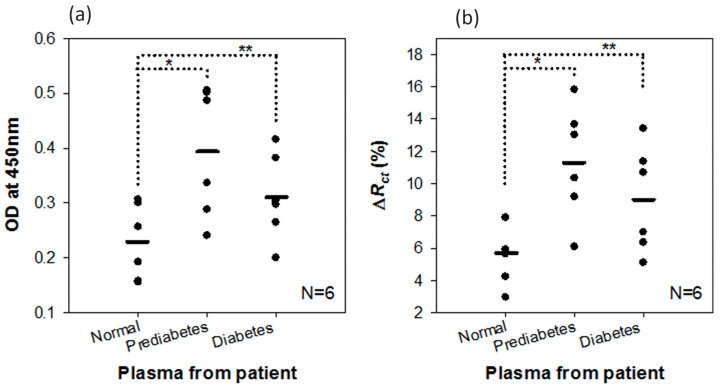
Analytical performance for the detection of NGAL: a commercially available ELISA NGAL detection kit (**a**) compared to sensor system from [230] (**b**). Statistical significance between groups was determined by ANOVA test, where *p* value is * *p* < 0.02 or ** *p* < 0.256 in (**a**), * *p* < 0.017 or ** *p* < 0.246 in (**b**), respectively. Mean values of each group are indicated with bold lines. Reprinted from [230] with permission of Elsevier provided by Copyright Clearance Center.

Peptide biosensors often do not require modification of electrodes using nanomaterials. An interesting example of an impedimetric peptide-based biosensor for detecting the S-protein of the SARS-CoV-2 virus is presented in [229]. The biosensor does not require nanomaterials or complex techniques but uses gold electrodes with a thiolated peptide whose interaction with protein S is measured by EIS. The biosensor showed high sensitivity and reproducibility, and the detection limit was 18.2 ng/mL for S-protein and 0.01 copies/mL for viral particles. The biosensor successfully identified protein S in samples from infected patients in 15 min without labels. The biosensor also has long-term stability (73.6% of the initial signal after 20 days of storage) and high repeatability and reproducibility (RSD 4.1% and 2.2%). Cho et al. [230] presented a biosensor for the detection of neutrophil gelatinase-associated lipocalin (NGAL), which is a marker of diabetic nephropathy. A peptide with the sequence DRWVARDPASIF, found using phage display, selectively bound to the target protein. The sequence was further modified with a cysteine at the C-terminus to form a self-assembled thiol monolayer, as well as a linker (-GGGGS-) for molecular flexibility on the gold surface. The affinity of the resulting peptide was measured using square wave voltammetry (SWV) and electrochemical impedance spectroscopy EIS. The LOD value by EIS was 1.74 ng/mL, and by SWV, it was 3.93 ng/mL. The performance of this biosensor is comparable to commercial ELISA tests, which was verified using real patient plasma samples (Figure 20).

**Figure 21:**
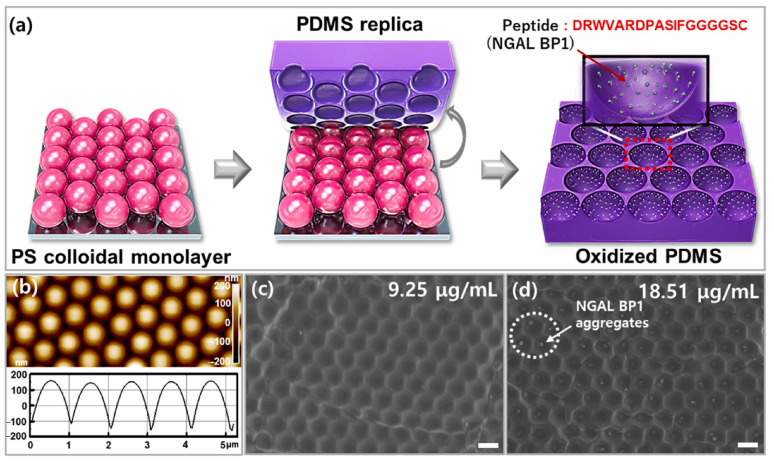
(**a**) Fabrication process of the (NGAL-BP1)-modified PDMS mold with hexagonally patterned pores for peptide surface imprinting via incubation in a peptide solution with a specific concentration. (**b**) Atomic force microscopy (AFM) image of the polystyrene (PS) colloidal monolayer and (**c**,**d**) scanning electron microscopy (SEM) images of oxidized PDMS molds with patterned concave pores after NGAL-BP1 adsorption during 2.5 h incubation in 9.25 and 18.51 μg/mL peptide solutions. All scale bars are 1 µm. Reprinted from [124] with permission of Elsevier provided by Copyright Clearance Center.

The improvement of this technique for detecting NGAL is presented in [124], where a sensor based on a film of molecularly imprinted polymer (MIP) with a specific binding peptide (BP1) was developed on a gold quartz electrode. The authors used a surface imprinting method with photopolymerization and a PDMS template with hemispherical depressions. Using EIS (Figure 21), the values of LOD 0.07 μg/mL and LOQ 0.24 μg/mL were achieved. They determined that the selectivity coefficient (k*) of NGAL protein on BP1 peptide-imprinted film was 3.5–5.8 relative to BSA over the entire low concentration range, indicating high affinity between BP1 peptide and NGAL protein.

Additionally, peptide-based dendrimers can be used to detect target proteins. Thus, Matsubara et al. [231] developed a biosensor to detect human and avian influenza virus (IFV) using gold electrodes with peptides that mimic receptors for viral hemagglutinins (HA). The authors modified the density and structure of peptides on boron-doped diamond (BDD) electrodes to improve virus binding and measure charge transfer resistance using electrochemical impedance spectroscopy. The ligands used were dendrimers having pentapeptide units mimicking the sialyl glycoconjugate receptor for uptake of IFV HA. The electrode with a peptide coating based on BDD had a fairly high sensitivity (3−400 pfu/sample), as did the electrode with antibodies based on BDD (5−10 pfu/sample).

### 4.4. Other Types of Biorecognition Elements Used in Impedimetric Biosensors for Protein Detection

***Enzymes.*** Enzymes are proteins that catalyze chemical reactions in living organisms. Enzymes can be used as recognition elements to detect their substrates, products, or inhibitors [232,233]. Enzymes have high specificity and activity and can also amplify the signal by generating a large number of reaction products. However, enzymes also have some disadvantages, such as being expensive, difficult to store, and susceptible to denaturation. However, impedance measurement is rarely used in enzyme-based biosensors compared to amperometry or potentiometry due to the relatively long time required to obtain the full impedance spectrum over a wide frequency range [234]. One of the most famous works in this direction is the biosensor from Chan et al. [235] for the determination of L-lactate based on a bioselective membrane of lactate dehydrogenase (LDH) and pyruvate oxidase (PyrOx). The biosensor is made by coating and crossing enzymes using glutaraldehyde vapor. The principle of operation of the biosensor is to measure the change in impedance caused by the formation of charged ions (CH_3_COO^−^, H^+^, and HCO_3_^−^) during the oxidation of pyruvate under the influence of PyrOx, pyruvate is formed during the oxidation of L-lactate under the action of LDH + nicotinamide adenine dinucleotide (NAD^+^). The detection limit was 17 and 20 μM for the resistance values at the interface between the two enzyme layers, R_IM_, and the corresponding capacitance, represented as CPE_IM_.

***Peptide nanotubes***. Peptide nanotubes are nanostructures that are formed from short peptides that self-assemble into tubular shapes [236]. Peptide nanotubes have unique electrical, mechanical, and biological properties that make them suitable for impedimetric biosensors. In [125], a biosensor was developed for the determination of interleukin 6 (IL-6), an important cytokine, based on a film of molecularly imprinted polymer with polydopamine (MIP(pDa)) on peptide nanotubes (PNT) deposited on screen-printed electrodes (SPEs). The authors used IL-6 as a template to self-polymerize dopamine without an initiator, enzyme, or crosslinker. They found that IL-6 could be detected in the range from 1 to 200 pg/mL with a good correlation between the redox couple and the logarithm of IL-6 concentration. The MIP(pDa)/PNT film had selective binding capacity for IL-6 with high recovery values in the urine sample.

**Figure 22:**
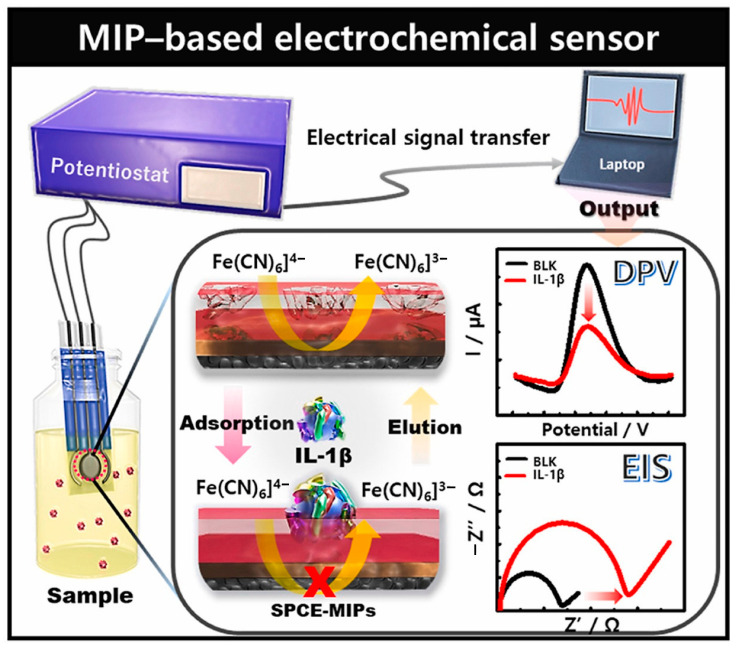
Molecularly imprinted polymer-based electrochemical impedimetric sensor for detection of trace cytokine IL-1β. Reprinted from [127] with permission of Elsevier provided by Copyright Clearance Center.

***Molecularly imprinted polymers***. Molecularly imprinted polymers are synthetic polymers that have specific molecular imprints matching the structure and size of the analyte [237]. They are also distinguished as a separate type of biorecognition element. Molecularly imprinted polymers can be prepared through an imprinting process where the analyte is used as a template for the polymerization of monomers and then removed from the polymer matrix. Molecularly imprinted polymers have high selectivity, stability, and regenerability and can also be quite easily synthesized and modified. Choi, D. Y. et al. [127] demonstrated a biosensor imprinted with IL-1β protein, a cytokine associated with inflammation, on printed carbon electrodes (SPCEs) (Figure 22). They deposited a bilayer of poly(o-phenylenediamine) and poly(chromotrope 2R) with IL-1β as a template using cyclic voltammetry (CV) to form an MIP film. The authors studied the electrochemical properties of the sensor using CV and electrochemical impedance spectroscopy (EIS) methods and confirmed the imprinting effect on the MIP film. They showed that the MIP film sensor has high sensitivity to trace amounts of IL-1β (several pg/mL) with an LOD of 0.23 pg/mL and LOQ of 0.78 pg/mL. Balayan et al. [165] developed an electrochemical biosensor for the detection of sepsis in newborns based on MIPs selective for serum amyloid A (SAA) protein. They coated the screen-printed electrode with multi-walled carbon nanotubes (MWCNTs), manganese oxide nanospheres (MnO_2_NSs), and cobalt oxide nanoparticles (Co_3_O_4_NPs) to obtain a synergistic effect and high conductivity. They then polymerized MIP, specifically synthesized for SAA, onto the modified electrode. The created biosensor operates in the range from 0.01 pM to 1 µM with a detection limit of 0.01 pM and has a fairly long service life (42 days).

***Affimers.*** Affimers are scaffolding proteins that are not antibodies but are capable of binding to other molecules with high affinity and specificity [238]. Affimers are obtained using the phage display method, in which those that have the highest affinity for the analyte of interest are selected from a library of random protein sequences [239]. Affimers have a small size (about 14 kDa) [240], high affinity (from subnano- to femtomolar binding constants), low immunogenicity, and the possibility of modification and functionalization. In addition, their advantage is their relatively low cost and ease of synthesis compared to antibodies. Shamsuddin et al. [105] presented an electrochemical biosensor for the detection of colorectal carcinoembryonic antigens (CEAs) based on polyoctopamine (POct), a nonconducting polymer with amine groups. The authors electropolymerized POct as a transmitter layer that allowed covalent binding of different bioreceptors, such as antibodies and synthetic affimer proteins, without surface activation. Affimers were fixed on the surface of gold screen-printed electrodes through the heterobifunctional crosslinking agent sulfo-SMCC (Sulfosuccinimidyl 4-(*N*-maleimidomethyl) cyclohexane-1-carboxylate). The close location of the bioreceptors to the transmitter layer significantly increased the sensitivity of detection. The sensitivity of the small bioreceptor (affimer, 12.6 kDa) to CEA was comparable to the large antibody (150 kDa) with a detection limit of 11.76 fM, much lower than the clinical level of 25 pM. However, the affimer-based biosensor had a narrower dynamic range than the immunosensor (1–100 fM vs. 1 fM–100 nM). All electrochemical measurements were performed in less than 5 min with a small sample volume (10 μL).

***Affibodies.*** Affibodies are engineered proteins that are produced from the Z domain of protein A from Staphylococcus aureus [241]. Affibodies have a small size (about 6 kDa), high thermal stability, salt resistance, and the ability to regenerate [242]. Affibodies can be synthesized using phage display or recombinant DNA technology [243]. Affibodies can be engineered to bind to a variety of target molecules, including proteins. Ravalli et al. [244] created an electrochemical biosensor for the detection of HER2, a cancer biomarker, based on affibodies, which serve as bioreceptors, and gold nanostructured printed graphite electrodes. They anchored a cysteine-terminated affibody onto gold nanoparticles. They showed that the disposable biosensor has good analytical performance for HER2 detection in the range of 0 to 40 μg/L with a detection limit of 6.0 μg/L.

**Figure 23:**
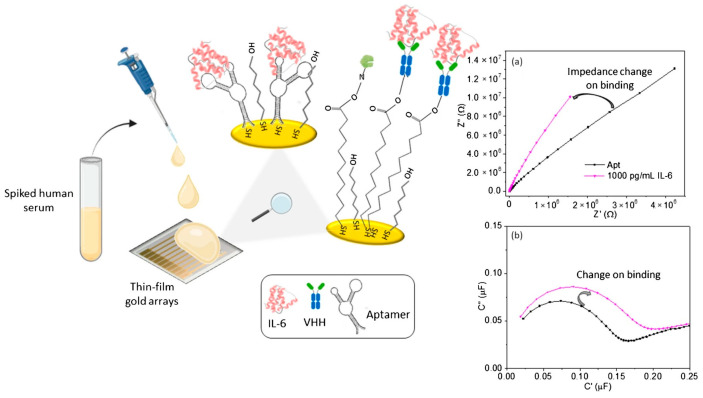
Schematic drawing of conceptual basis of IL-6-recognizing biosensor. Gold electrodes are modified with a nanobody or an aptamer specifically recognizing IL-6. There is a change in the non-faradaic impedance spectra when these surfaces are challenged in serum samples containing IL-6, which can be observed in both impedance (**a**) and capacitance planes (**b**). Reprinted from [247], license CC BY-NC-ND 4.0.

***Nanobodies.*** Nanobodies are antibody fragments consisting of one monomeric variable domain of the antibody heavy chain [245,246]. They have a high ability to bind antigens and are the smallest functional fragments derived from natural immunoglobulins. Nanobodies are found in animals of the camelid family, in which the antibodies do not contain a light chain. Nanobodies have unique physicochemical and structural characteristics that make them ideal candidates for the development of diagnostic tests. Sánchez-Salcedo et al. [247] presented an electrochemical biosensor for the detection of IL-6 (Figure 23). The authors used a cheap, thin, eight-channel gold sensor array on a flexible substrate as electrodes. They used gold surfaces modified with an anti-IL-6 nanobody (anti-IL-6 VHH) or a specific IL-6 aptamer. In the first system, the nanobody was covalently linked to the gold surface using a self-assembled bilayer monolayer of 6-mercapto-1-hexanol (MCH) and 11-mercaptoundecanoic acid. In the second system, the aptamer was chemically adsorbed onto the electrode surface in a mixed monolayer with MCH. Using nanobodies, the authors detected IL-6 in the range from 10 pg/mL to 10 ng/mL. They noticed that the capacitance of the electrodes drifted monotonically during prolonged incubation in the buffer. This could mask a specific response, especially in a wearable format where long measurement times are required. Using aptamers, the authors achieved the detection of IL-6 in the range from 10 to 10,000 pg/mL. In contrast to the nanobody-based platform, the aptasensor was less susceptible to background drift, which stabilized after 90 min.

The work [248] describes two nanobiocomposite platforms based on synthetic nanobodies, which allow label-free electrochemical detection of epidermal growth factor receptor (EGFR). To do this, either NiO particles or a layer of poly(thiophene acetic acid) (PTAA) are applied to screen-printed carbon electrodes, to which an EGFR-specific nanobody with dual functionality—a 6xHis tag and lysine—is attached. These nanobodies serve as bioreceptors for impedance sensing in the presence of EGFR. The platforms are characterized by various methods and show good sensitivity and specificity for EGFR ranging from 0.25 to 50 μg/mL with detection limits of 0.46 μg/mL and 1.14 μg/mL, respectively.

Impedimetric biosensors can be used to detect and quantify various proteins, which are important biomarkers for the diagnosis, monitoring, and treatment of various diseases such as cancer, infections, inflammation, allergies, etc. However, impedimetric biosensors also face several problems and limitations, such as instability, degradation, nonspecific binding, interference, drift, etc. Therefore, the development and implementation of new types of recognition elements that can improve the performance, reliability, and functionality of impedimetric biosensors is an urgent and promising task.

## 5. Integration of Impedimetric Biosensors into Microfluidic Systems

In addition to providing high sensitivity and selectivity, modern biosensor systems must provide rapid detection, be cost-effective, and also be miniaturized, portable, and have small sample volumes. Problems associated with miniaturization, namely reducing the volume of the analyte and increasing analysis productivity, are of significant interest from the point of view of using methods and approaches of microfluidics to solve them. Microfluidics is a field of science and technology that studies and controls the flow of liquids on a microscale [249,250]. Microfluidics is of great importance in integration with biosensor systems, as it allows the manipulation of biological samples and reagents with high precision, efficiency, and speed. Microfluidic systems can integrate microchannels, micropumps, microshutters, micromixers, microreactors, and other elements [251,252,253], which can be made of various materials, such as silicone, polymers, glass, metals, etc. [254,255,256].

Integrating impedimetric biosensors with microfluidic systems provides several advantages, such as (i) reduced consumption of biological samples and reagents, which reduces cost and risk of contamination; (ii) increasing the sensitivity and selectivity of biosensors, as microfluidics provides better control over mass transport, temperature, pH, etc.; (iii) faster analysis and reaction times as microfluidics reduces diffusion distance and mixing time; and (iv) the ability to automate and integrate various bioanalytical functions such as sample preparation, separation, concentration, detection, etc., in a single microfluidic platform (e.g., lab-on-a-chip).

In addition, microfluidics is facilitating the development of point-of-care (POC) impedimetric biosensor systems that can be used to quickly, easily, and accurately diagnose and monitor various diseases at the point of patient care, such as at home or in the clinic [257,258,259].

There are various examples of the use of impedimetric biosensors in combination with microfluidic systems for protein detection [260,261,262,263,264,265]. Alsabbagh et al. [266] present a microfluidic circuit for electrochemical impedance spectroscopy (EIS), which can serve as a biosensor for label-free detection of cardiac troponin I. The biosensor system consists of a glass plate, sputtered electrodes, and a polydimethylsiloxane (PDMS) microchannel. Functionalization methods have been developed for electrodes that take into account the possible charge transfer through the sensitive layer, the specific binding of the analyte to the corresponding antibodies, and the reduction in nonspecific protein adsorption. The use of a 1000 ng/mL human serum albumin (HSA) sample did not result in a noticeable change in impedance, while the use of a 1 ng/mL cardiotroponin I sample caused a significant shift in the Nyquist plot.

**Figure 24:**
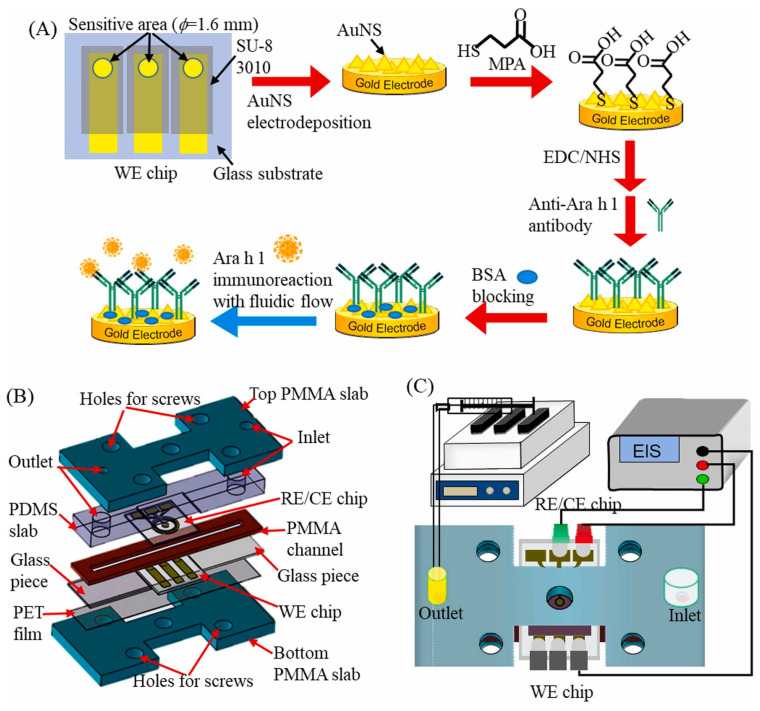
The schematic illustration of the immunosensor preparation based on gold nanostructure (AuNS) electrodeposition followed by 3-mercaptopropionic acid (MPA) modification, antibody immobilization, and BSA blocking (**A**). The design and assembly of a microfluidic device integrating top–bottom opposite electrodes (**B**). The device was connected to a syringe pump for controlling the flow rate and an electrochemical apparatus for EIS measurement (**C**). Reprinted from [267] with permission of Elsevier provided by Copyright Clearance Center.

Wu et al. [267] developed a microfluidic device with two opposing electrodes and a channel to create immunosensors for the peanut allergen Ara h 1 (Figure 24). With optimal fluid management, the device can obtain a better immune response by pumping 80 μL of Ara h 1 solution supplemented with 2.5 mM Fe(CN)_6_^3−/4−^ mediator at a rate of 50 μL/min for 2.5 min. A single pass of the sample through the microfluidic system provides a linear detection range of 1 pg/mL–10 ng/mL and an LOD of 3.9 fg/mL, respectively. The developed device can determine the Apa h 1 concentration with good recovery power (95−103%) for a diluted supernatant isolated from standard peanut butter. The microfluidic system creates a sealed sample chamber with a predetermined volume to ensure standardization of measurements. Bhardwaj et al. [268] presented an aptamer-based microfluidic biosensor for in-line monitoring of ranibizumab in bioreactors. The aptamer with the best affinity for the analyte was immobilized on gold microelectrodes on a microfluidic chip, which was fabricated using a glass base and PDMS top. The linear detection range and detection limit were 25–100 nM and 25 nM, respectively, which were significantly better than the HPLC-based detection method (about 240 nM). The device did not require preconcentration or sample pretreatment and resulted in detection times of about 30 min compared to several hours for HPLC.

**Figure 25:**
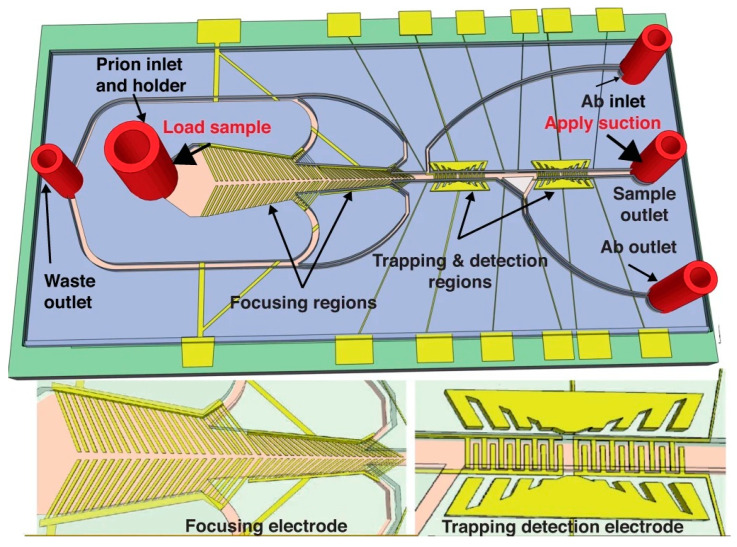
A 3D view of the biosensor CHD for diagnosis. Reprinted from [271], license CC BY-NC-ND 4.0.

Using microfluidic systems makes it convenient to combine several different functional zones in one device [269,270]. Muhsin et al. [271] present a microfluidic biosensor device based on a microelectromechanical system (MEMS) for detecting pathological prions of chronic wasting disease (CHD) in deer. The device consists of functional zones (Figure 25) for the concentration, capture, and detection of the prion. The detection zone contains an array of electrodes coated with a monoclonal antibody against pathological prions. Testing can be completed in less than 1 h with high sensitivity and selectivity. The biosensor detected the engineered prion antigen at a dilution of 1:24, while the ELISA detected the same antigen at a dilution of 1:8. The relative detection limit of the biosensor was 1:1000 dilution of a known strong positive sample, while the ELISA showed a 1:100 dilution.

In addition, microfluidics allows the creation of multiplex impedimetric biosensor systems that can simultaneously detect and measure multiple targets in a single sample [272,273,274]. This improves the information content and reliability of the analysis and also saves time and resources. Multiplex impedimetric biosensors can be implemented by dividing the electrodes into different regions, each with its own receptor, or by using different frequencies to excite and measure impedance on a single electrode with multiple receptors. However, there is a fairly small number of works in the direction of multiplex microfluidic impedance biosensing. Article [275] presents a 2 × 4 interdigitated electrode (IDE) array to improve the sensitivity of immunoassays using microparticles as a label. To amplify the signal, the system is integrated into a microfluidic channel. The developed impedimetric biosensor includes all the necessary components for bioassays in one system, which consists of a gold chip with an IDE array, an eight-channel impedance analyzer with software for semi-real-time data acquisition, and a PDMS microfluidic chip for sample delivery and washing. Therefore, the creation of microfluidic chips for impedance detection of several proteins at once is a relevant and important task. This will help to create cheap biosensor panels for point-of-care diagnostics of a number of diseases.

## 6. Conclusions

Recently, the biosensor market has expanded significantly due to the increased demand for specialized sensors that can provide fast and accurate results in various scientific fields. In this article, we reviewed the basic principles, methods, and applications of impedimetric biosensors for protein detection. The material demonstrated that these biosensors have several advantages, such as simplicity, speed, sensitivity, selectivity, cost-effectiveness, and the ability to be integrated into microfluidic systems. Impedimetric biosensors represent a powerful and promising tool for studying and monitoring biological processes associated with proteins and can contribute to the development of new approaches to the diagnosis and treatment of various diseases.

The impedance measurement technique is one of the important factors in the creation of impedimetric biosensors. Impedimetric biosensors can be faradaic or non-faradaic, depending on the presence or absence of a redox-active probe in the solution. Non-faradaic biosensors may be simpler for practical use since they do not require additional electrodes or the addition of a redox couple and can operate at a single frequency. However, faradaic biosensors can demonstrate greater analytical sensitivity.

An important goal is to improve the operational characteristics of impedimetric biosensors. Its solution requires a deep understanding of the properties of materials for creating and functionalizing electrodes and methods for their manufacture. Current analysis identifies the main groups of materials that are widely used for the formation of electrodes of impedimetric biosensors and their modification. The main materials used to create electrodes are gold (Au), carbon (graphite), and conductive metal oxides. Electrodes based on Au and carbon materials are typically formed by screen printing together with a counter electrode and a reference electrode. This technology is highly scalable for mass production. It is also compatible with the use of substrates (for example, PET) with coatings of conductive metal oxides (for example, ITO), including the possibility of additional irradiation of the sensor from the substrate. Screen-printing electrodes are modified with various nanomaterials, such as graphene, carbon nanotubes, polymers, gold nanoparticles, metal oxide nanoparticles, quantum dots, and others. The sensor platforms with screen-printed electrodes and modifying nanomaterials for faradaic EIS, as well as interdigitated electrodes for non-faradaic EIS, are currently commercially available. This certainly contributes to an increase in the amount of research in the field of impedimetric biosensors, their clinical trials, and their applications. However, research into the application of various nanomaterials, new polymers, and biopolymers, including MIPs, as well as various molecular structures, remains urgently relevant.

The need and choice of electrode-modifying materials are primarily determined by the choice of the linker used or the immobilization method (covalent binding or physical adsorption) and biorecognition element, biocompatibility, as well as the required response range and detection limit to values significant for clinical studies. Thus, obviously, for many biomarkers, obtaining the required wide range of analytical responses is ensured by increasing the specific electrode area with nanomaterials, nanostructures, and the use of composite materials. New modifying materials, their decoration, and the creation of compositions based on them also help to amplify the signal to the required sensitivity. Also, the choice of electrode materials is determined by the ease of its synthesis and implementation in the sensor system, the ability to scale the technology, and its durability. As we can see from the review, the use of new materials to create impedimetric biosensors looks promising.

The selection of the required highly specific biorecognition elements and the technology for their immobilization is also an important component of modern impedimetric biosensors. The choice of the bioligand immobilization method depends on the specific goals and conditions of the analysis, as well as on the type of biorecognition element and transducer. In some cases, it may be advisable to use a combination of different immobilization methods to achieve the optimal steric configuration of the biorecognition element. In this way, the probability of affinity binding of the ligand to the target protein can be significantly improved. Along with traditional antibodies, aptamers, and enzymes, the popularity of biosensors based on peptides, as well as other new bioligands, is increasing. The key challenge in creating new biorecognition elements is to achieve high sensitivity and selectivity while achieving low-cost mass production to reduce the overall cost of the analysis.

Integration of impedimetric biosensors with microfluidic systems reduces sample volume and detection time and also holds great promise for creating point-of-care systems that can be portable and used in emergency care. To do this, it is necessary to create new biosensors for multiplex determination of various protein markers, which can be implemented in the format of a miniature chip.

The creation of impedimetric biosensors for protein detection is a complex and multifaceted task that includes various aspects, including the following:

  (i). Selection and synthesis of suitable materials and nanostructures for electrodes that should provide good electrochemical activity, resistance to corrosion and degradation, and the ability to functionalize the surface for immobilization of bioligands.
 (ii). Development and optimization of methods for immobilizing biorecognition agents on the surface of electrodes, which should ensure specific, stable, and oriented binding to target proteins, as well as preservation of their activity and structure.
(iii). Improving the specificity and sensitivity of biosensors, which should be able to detect low concentrations of proteins in complex biological matrices such as blood, serum, plasma, saliva, and urine, as well as distinguish between proteins with high degrees of homology or post-translational modifications.
 (iv). Reduction of the influence of interference and nonspecific binding, which can distort the biosensor signal and degrade its performance. Interference can be caused by electromagnetic fields, temperature, pH, salt composition, oxidizing or reducing agents, and other biomolecules present in the sample.
  (v). Adaptation of biosensors to real samples and operating conditions, which may differ from laboratory ones. For example, biosensors must be able to operate in a wide range of temperatures, humidities, pressures, and illuminations and also be resistant to mechanical damage and contamination.
 (vi). Expanding the range of detectable proteins that can be associated with various diseases, such as cancer, infections, autoimmune diseases, allergies, and others. To achieve this, it is necessary to develop new biorecognition agents that can specifically bind to proteins of interest and also take into account their structural and functional characteristics.

Indeed, the task of creating new effective biosensor systems is complex and multifactorial. To solve it successfully, it is necessary to apply an interdisciplinary approach, combining knowledge and skills in the fields of chemistry, physics, biology, materials science, nanotechnology, electronics, computer science, and medicine.

## Figures and Tables

**Table 1:** Examples of impedimetric biosensors based on metal electrodes for protein detection.

Electrodes	Biorecognition Element	Target Protein	Matrix	Sensitivity	Refs.
Au-IDWμE	IgG-Fc fragment	IgM-RF	Human serum	10–200 IU/mL range; LOD = 0.22 IU/mL	[57]
Au	anti-NS1 antibody	NS1	Neat serum	0.01–2.00 µg/mL range; LOD = 3 ng/mL	[58]
Au	anti-N antibody	N protein of SARS-CoV-2	Human saliva	4.4 ng/mL–4.4 pg/mL range; LOD = 0.362 ng/mL	[59]
Au	anti-human CRP antibody	C-reactive protein	Human serum	0.5–50 nM range; LOD = 176 pM	[60]
Au-IDEs; Au-Colloidal Au NPs	antigen tTG	αtTG-Abs	Serum	30 pM–30 nM range	[61]
Au	IgG-antibody	IgG	PBS buffer	0.9–50 mg/L range	[62]
Au	anti-tau antibody	Tau	Human serum	10^−14^–10^−7^ M range; LOD = 0.03 pM	[63]
Au	aptamer	N-terminal pro b-type natriuretic peptide	Artificial human saliva	5.0 × 10^−3^–1.0 pg/mL range; LOD = 5.0 × 10^−3^ pg/mL	[64]
Au	anti-A1BG antibody	A1BG	Human serum	1–300 ng/mL range; LOF = 1 ng/mL	[65]
Au	DNA aptamers	NS1	Human serum	0.025 ng/mL	[66]
Au	anti-A1AT antibody	A1AT	Artificial serum	100–600 μg/mL range; LOD = 11.9 μg/mL	[67]
Au	anti-SPB antibody	SPB	Amniotic fluid	2–2000 ng/m L range; LOD = 0.1 ng/mL	[68]
Au/Au NPs	aptamers anti-PSA and PSAG-1	PSA	Human serum	0.26–62.5 ng/mL (PSAG-1), 0.64–62.5 ng/mL (anti-PSA) range	[69]
Carbon; Au NPs	aptamer Gli4-T	PWG-Gliadin	Buffer	0.1–1 mg/L of gliadin range; LOD = 0.05 mg/L	[71]
Glassy carbon/Au NPs	aptamer	IL-6	Human serum	5 pg/mL–100 ng/mL range; LOD = 1.6 pg/mL	[72]

**Table 2:** Examples of impedimetric biosensors based on conducting oxides for protein detection.

Electrodes	Biorecognition Element	Target Protein	Matrix	Sensitivity	Refs.
ITO	anti-IL-8 antibody	IL-8	Human serum and saliva	0.02–3 pg/mL range; LOD = 6 fg/mL	[73]
FTO	anti-IL-1α antibody	IL-1α	Human serum and saliva	0.02–2 pg/mL range; LOD = 6 fg/mL	[74]
ITO	anti-MAGE-1 antibody	MAGE-1	Human serum	4–200 fg/mL; LOD = 1.30 fg/mL	[75]
ITO	anti-PAK 2 antibody	PAK 2	Human serum	0.005–0.075 pg/mL range; LOD = 1.5 fg/mL	[76]
ITO	anti-PAK2 antibody	PAK-2	Artificial and real human serum	0.05–2.5 pg/mL range; LOD = 0.0252 pg/mL	[77]
ITO	anti-Aβ_42_ antibody	Aβ_42_	Human serum	1–100 pg/mL range; LOD = 0.37 pg/mL	[78]
VASiNWs/ITO	antibody (cTnIAb)	cTnI	Human blood sera	1.76011–17,601.1 ng/mL range; LOD = 0.53 ng/mL	[79]

**Table 3:** Examples of impedimetric biosensors based on carbon materials for protein detection.

Electrodes	Biorecognition Element	Target Protein	Matrix	Sensitivity	Refs.
Carbon (SPCE)	bicyclic peptides	h-uPA	Buffer	0.01–1 ng/mL range; LOD = 9 ng/mL	[80]
Carbon nanofibers	aptamer-LYS	Lysozyme (Lys)	Fetal bovine serum	LOD = 0.36 μg/mL	[81]
MWCNTs	anti-Mb-IgG antibody	Mb	Human serum	0.1–90 ng/mL range; LOD = 0.08 ng/mL	[82]
MWCNTs	thrombin aptamer	α-thrombin	5 mM [Fe(CN)_6_]^3−/4−^ in PBS	0.39–1.95 nM range; LOD = 105 pM	[83]
Carbon nanotube	anti-lysozyme DNA aptamer	LYS	2.5 mM [Fe(CN)_6_]^3−/4−^ in KCl	12–400 µg/mL range; LOD = 12.09 µg/mL	[84]
MWCNT	anti-IL-8 antibody	IL-8	PBS	1–1000 pg/mL range; LOD = 0.1 pg/mL	[85]
MWCNT/AuNPs	anti-DJ-1antibody	DJ-1	Cerebrospinal fluid and saliva	4.7–4700 fg/mL range; LOD = 0.5 fg/mL	[86]
AuNPs@MWCNTs-GQDs	anti-PSA antibody	PSA	[Fe(CN)_6_]^3−/4−^ in KCl	1–10,000 pg/mL range; LOD = 0.48 pg/mL	[87]
Glassy carbon; boron-doped diamond	anti-N antibody	N protein of SARS-CoV-2	Human saliva	4.4 ng/mL–4.4 pg/mL range; LOD = 0.227 ng/mL (glassy carbon) and 0.334 ng/mL (boron-doped diamond)	[59]
C_60_-modified graphite paper	anti-ST2 antibody	ST2	Human serum	0.1–100 fg/mL range; LOD = 0.124 fg/mL	[88]
GP	anti-adiponectin antibody	Adiponectin	Human serum	0.05–25 pg/mL range; LOD = 0.0033 pg/mL	[89]
GP/Au NPs	anti-CK antibody	Creatine kinase (CK)	Human blood	0.1–50 pg/mL range; LOD = 0.045 pg/mL	[90]
Boron-doped diamond	anti-S1 antibody	Subunit S1 of SARS-CoV-2	Complex matrix	1 fg/mL	[91]
Gii-sense^®^ Graphene Foam	anti-IL-10 antibody	IL-10	Artificial saliva	10–100 fg/mL range; LOD = 7.89 fg/mL	[92]
ErGO	lectin concanavalin A	Glycoprotein invertase (INV)	PBS	10^−14^–10^−8^ mol range	[93]
rGO/AuNPs	anti-Tau antibody	Tau-441	Serum and cerebrospinal fluid (CSF)	1–500 pg/mL range; LOD = 0.091 pg/mL	[94]
Porous graphene oxide (PrGO)	anti-cTnI antibody	cTnI	Human blood	0.1–10 ng/mL range; LOD = 0.07 ng/mL	[95]
rGO	anti-CEA antibody	CEA	Human blood serum	0.1–5 ng/mL range; LOD = 0.05 ng/mL	[96]
AuNP electrode/GO	anti -HSP-47 antibody	HSP-47	Cell lysates	10–160 pg/mL range; LOD = 9.47 pg/mL	[97]
rGO-NP	anti-CRP antibody	CRP	PBS, Human serum	1–1000 ng/mL range; LOD = 0.06 ng/mL (in PBS) and 0.08 ng/mL (in human serum)	[98]
Maze-like boron-doped carbon nanowall	anti-protein D antibodies	Protein D	PBS	3.37 × 10^−11^–3.37 × 10^−3^ µg/mL range; LOD = 2.39 × 10^2^ fg/mL	[99]
HsGDY@NDs	aptamers	cTnI; Myo	PBS	LOD = 6.29 fg/mL (cTnI); 9.04 fg/mL (Myo)	[100]

**Table 4:** Examples of impedimetric biosensors based on polymeric materials for protein detection.

Electrodes	Biorecognition Element	Target Protein	Matrix	Sensitivity	Refs.
Poly ortho-phenylenediamine	mAβ antibody	Aβ40	PBS	1 pg/mL	[101]
Polycarboxybetaine methacrylate (pCBMA)	anti-IL-8 antibody	IL-8	Nasal epithelial lining fluid	55 fM–55 nM range; LOD = 10 fM	[102]
Polytyramine	anti-FGFR3-14 and FGFR3-21 affimer proteins	FGFR3	PBS and synthetic urine	sub-pM–nM range	[103]
Copolymer of polytyr-amine/3-(4-hydroxyphenyl) propionic acid (POPA)	fragment of the cellular prion protein (PrPC, residues 95–110)	AβO	PBS	10^−12^–10^−6^ M range; LOD~0.5 pM; (Aβ peptide)	[104]
Polyoctopamine	CEA affimer; anti-CEA antibody	CEA	Spiked human serum	1–100 fM (affimer) and 1 fM–100 nM (antibody) range; LOD = 11.76 fM	[105]
Polypyrrole polymer	IL-6 receptor	IL-6	Human serum	0.02–16 pg/mL range; LOD = 6.0 fg/mL	[106]
Poly-pyrrole-pyrrole 3 carboxylic acid	antibodies (Ab-PfHRP2)	PfHRP2	[Fe(CN)_6_]^4−/3−^+ KCl solution	100–1000 ng/mL range; LOD = 27.47 ng/mL	[107]
Carboxy-endcapped conductive polypyrrole	anti-GPC3 antibody	gypican-3 (GPC3)	Human serum	0.9 pg/mL to 9 ng/mL range; LOD = 0.3 pg/mL	[108]
Acid-substituted poly(pyrrole)	anti-CC chemokine receptor 4 (CCR4) antibodies	CCR4	Human serum	0.02–8 pg/mL range; LOD = 6.4 fg/mL	[109]
AuNPs/thiophene polymer P(ThiAmn)	anti-GM2A antibodies	GM2A	Human serum	0.0185–111 pg/mL range; LOD = 5.8 fg/mL	[110]
acetylene black (AB)/epoxy-substituted-poly(pyrrole) polymer	anti-IL-6 antibodies	IL-6	Spiked human serum	0.01–50 pg/mL range; LOD = 3.2 fg/mL	[111]
Overoxidized polypyrrole decorated with gold nanoparticle	anti-HigG antibody	HigG	Human serum	0.5–125.0 ng/mL range; LOD = 0.02 ng/mL	[112]
Methyl orange-doped polypyrrole	DMIP	CEA; AFP	Human serum	5–10^4^ pg/mL (CEA), 10–10^4^ pg/mL (AFP) range; LOD = 1.6 pg/mL (CEA), 1.6 pg/mL (AFP)	[113]
Acetylene black/thiophene polymer	anti-RBD antibody	spike receptor (RBD) COVID-19	Nasal secretions	0.0012–120 pg/mL range; LOD = 0.58 fg/mL	[114]
Polyaniline (PANI)	IFN-γ antibody	IFN-γ	Human serum	5–1000 pg/mL range; LOD = 3.4 pg/mL	[115]
(PEDOT)/Au NP composites	anti-VEGF antibody	VEGF	[Fe(CN)_6_]^4−/3−^+ KCl solution	1–20 pg/mL range; LOD = 0.5 pg/mL	[116]
Silk protein with PEDOT:PSS	anti-VEGF_165_ antibodies	VEGF	Human serum	1 pg/mL–1 μg/mL range; LOD = 1.03 pg/mL	[117]
Carbon black C45/polythiophene polymer	anti-CYFRA 21-1 antibody	CYFRA 21-1	Human serum	0.03–90 pg/mL range; LOD = 4.7 fg/mL	[118]
Acrylamide (and Fe_3_O_4_ NP decorated with MWCNT-GO composite)	MIP + anti-PSA antibody	PSA; Myo	Human serum and urine	0.01–100 ng/mL (PSA), 1–20,000 ng/mL (Myo) range; LOD = 5.4 pg/mL (PSA), 0.83 ng/mL (Myo)	[119]
Polyglutamic acid (PGA)	anti-leptin antibody	leptin	[Fe(CN)_6_]^4−/3−^+ PBS solution	0.2–20 pg/mL range; LOD = 0.00813 pg/mL	[120]
Biotinylated phenylalanine nanoparticle	Asp-Glu-Val-Asp (DEVD)-containing peptide	caspase-3	[Fe(CN)_6_]^4−/3−^ solution	26 pM–125 pg/mL range	[121]
Gelatin	anti-SARS-CoV-2 Spike glycoprotein S1 antibody	SARS-CoV-2 spike glycoprotein S1	PBS and nasopharyngeal fluid	0.001–10 µg/mL range; LOD = 169 pg/mL (in PBS), 90 pg/mL (in nasopharyngeal fluid)	[122]
3-Aminophenol polymer	MIP	Tau protein	PBS	2.18 pM–2.18 nM range; LOD = 0.02 pM	[123]
Binding peptide (BP1)-imprinted film	MIP	neutrophil gelatinase-associated lipocalin	PBS	LOD = 0.07 μg/mL	[124]
Polydopamine	MIP	IL-6	[Fe(CN)_6_]^4−/3−^+ PBS solution	1–200 pg/mL range; LOD = 0.25 pg/mL	[125]
NiO NP/ePDA	MIP	Trp	[Fe(CN)_6_]^3−/4−^ solution	1–90 pg/mL range; LOD = 0.75 pg/mL	[126]
Poly(chromotrope 2R) (C2R)	MIP	IL-1β	PBS solution	LOD = 0.23 pg/mL	[127]
Au NPs conjugated with Dihexadecylphosphate	mAb-DD antibody	D-dimer (DD)	PBS	LOD = 8.92 ng/mL	[128]
PAMAM generation 2	DNA aptamer	APC	[Fe(CN)_6_]^4−/3−^+ KCl solution; fetal bovine serum	0.74–7.5 range; µg/mL LOD = 0.74 µg/mL (in buffer), 2.03 µg/mL (in serum)	[129]
PAMAM generation 2	DNA aptamer	APC	[Fe(CN)_6_]^4−/3−^+ KCl solution; fetal bovine serum	1–2.5 µg/mL; LOD = 1.81 µg/mL (in buffer), 0.02 µg/mL (in diluted fetal bovine serum)	[130]
Fullerene–PAMAM(G5) composite	anti-Fetuin-A antibody	HFA	Real blood samples	1.66–134 ng/mL range; LOD = 0.48 ng/mL	[131]

**Table 5:** Examples of impedimetric biosensors based on metal complexes of porphyrins, phthalocyanines, and metal–organic frameworks for protein detection.

Electrodes	Biorecognition Element	Target Protein	Matrix	Sensitivity	Refs.
Cu-MOF	Ab2-CA15-3 antibody (and Ab1 on Au NPs)	CA15-3	[Fe(CN)_6_]^4−/3−^+ KCl solution	10 μU/mL–10 mU/mL and 10 mU/mL–100 U/mL ranges; LOD = 5.06 μU/mL for CA15-3	[132]
Cu_3_(BTC)_2_ (copper-MOF)/PANI composite	anti-cTnI antibodies	cTnI	[Fe(CN)_6_]^4−/3−^+ PBS solution	1–400 ng/mL range; LOD = 0.8 ng/mL	[133]
FeCo-MOF/nickel foam	IL-1RA antibodies	IL-1RA	PBS and human serum	10 fg/mL–10 ng/mL range; LOD = 7.30 fg/mL (in buffer), 7.22 fg/mL (in serum)	[134]
Au@UiO-66-NH_2_	DNA biomimetic clamp	2 nucleocapsid protein (SARS-CoV-2 antigen)	Saliva and serum	LOD = 0.31 pg/mL	[135]
Zr(III) tetra(4-aminophenyl) porphyrin (TAPP) complex	antibody of NSE	NSE	[Fe(CN)_6_]^4−/3−^+ PBS solution	10.0 fg/m–2.0 ng/mL range; LOD = 7.1 fg/mL	[136]
Co phthalocyanines–Ce oxide NP conjugate	HB5 aptamer	HER2	Human serum	1–10 ng/mL range; LOD = 0.2 ng/mL	[137]
Cobalt-based phthalocyanine/graphene quantum dots	HB5 aptamer	HER2	Human serum	LOD = 0.0027 ng/mL	[138]

**Table 6:** Examples of impedimetric biosensors based on metal oxides and dielectrics for protein detection.

Electrodes	Biorecognition Element	Target Protein	Matrix	Sensitivity	Refs.
Au NPs/TiO_2_ NPs	DNA aptamer	leptin	Human serum	1.0–100.0 pg/mL and 100.0–1000.0 pg/mL ranges; LOD = 0.312 pg/mL	[139]
Polymer/TiO_2_ NPs	protein A	IgG	[Fe(CN)_6_]^4−/3−^+ KCl solution	0.0062–500 µg/mL range; LOD = 0.57 ng/mL	[140]
Titanium dioxide nanotube (TNT) array	anti-IL-6, anti-IL-8, and anti-TNFα AB	IL-6, IL-8, TNFα	PBS	5–2500 pg/mL range; LOD = 5 pg/mL	[141]
AuNPs/WO_3_/CNTs	SARS-CoV-2 antibodies	SARS-CoV-2-S protein	[Fe(CN)_6_]^4−/3−^ in buffer solution	0.125–16.0 pg/mL ranges; LOD = 1.8 pg/mL	[144]
Tungsten trioxide nanosheets (WO_3_ NS)	anti-cTnI antibodies	cTnI	[Fe(CN)_6_]^4−/3−^+ PBS solution	0.1–100 ng/mL range	[145]
ZnO/CuO composite nanoparticle	anti-cas9 antibody	caspase-9	Buffer solution	0.1–1 U/mL range; LOD = 0.07 U/mL	[51]
Silicon nitride	anti-HSA antibodies	HSA	PBS	10^−13^–10^−7^ M range; LOD = 10^−14^ M	[146]
Glass	anti-HSA antibodies	HSA	PBS	LOD = 2 × 10^−4^ mg/mL	[147]
SiO_2_	anti-pLDH Antibody	pLDH	Saliva	LOD = 250 pg/mL	[148]
ZnO thin film	antibodies	PCT, CRP	Human serum and blood	0.01–10 ng/mL (PCT), 0.01–20 µg/mL (CRP), ranges; LOD = 0.10 ng/mL (PCT); 0.10 µg/mL (CRP)	[149]
ZnO–CuO composite	anti-CRP antibodies.	CRP	PBS	1–10 ng/mL range	[150]
Nano-ZnO/CuO membranes	anti-CRP antibodies	CRP	PBS	0.1–15 ng/mL range; LOD = 16 pg/mL	[151]

**Table 7:** Examples of impedimetric biosensors based on other materials for protein detection.

Electrodes	Biorecognition Element	Target Protein	Matrix	Sensitivity	Refs.
L-cysteine-SnTeSe QD	interferon-gamma aptamer	interferon-gamma	Serum samples	10–55 pg/mL range; LOD = 0.151 pg/mL	[152]
Carboxylated CdTe QDs	anti-EP DIII ZIKV antibodies	EP-ZIKV	[Fe(CN)_6_]^3−/4−^ + buffer	LOD = 0.1 ng/mL	[153]
MoS_2_ nf	anti-TNF-α antibodies	TNF-α	Serum samples	1–200 pg/mL range; LOD = 0.202 pg/mL	[154]
AuNPs/Ti_3_C_2_-MXenes	aptamers	cTnI, Myo	[Fe(CN)_6_]^3−/4−^ + buffer	0.24 fg/mL–24 ng/mL (cTnI), 1–72 ng/mL (Myo) ranges; LOD = 0.14 fg/mL (cTnI), 0.2 ng/mL (Myo)	[155]
Au-NPs decorated MoS_2_ nanosheet	C-RP antibody	C-RP	Human serum	1 fg/mL–1 μg/mL range; LOD = 0.01 fg/mL	[156]
MP@silica@Au/CB	anti-fPSA antibody (and lectin (SNA-I) for Au-electrode/CB)	PSA	Spiked human serum	0.01–1 pg/mL range; LOD = 1.2 fM	[157]
Magnetic nanoparticles	anti-CRP polyclonal antibody (and anti-CRP monoclonal antibody on electrode)	CRP	PBS	10–200 ng/mL range; LOD = 0.34 ng/mL	[158]

## Data Availability

Not applicable.
